# *Arabidopsis* ANGULATA10 is required for thylakoid biogenesis and mesophyll development

**DOI:** 10.1093/jxb/eru131

**Published:** 2014-03-24

**Authors:** Rubén Casanova-Sáez, Eduardo Mateo-Bonmatí, Saijaliisa Kangasjärvi, Héctor Candela, José Luis Micol

**Affiliations:** ^1^Instituto de Bioingeniería, Universidad Miguel Hernández, Campus de Elche, 03202 Elche, Alicante, Spain; ^2^Department of Biochemistry and Food Chemistry, University of Turku, FI-20014 Turku, Finland

**Keywords:** *Arabidopsis thaliana*, chloroplast, grana, LHCII trimers, mesophyll development, thylakoid biogenesis, thylakoid stacking.

## Abstract

The chloroplasts of land plants contain internal membrane systems, the thylakoids, which are arranged in stacks called grana. Because grana have not been found in Cyanobacteria, the evolutionary origin of genes controlling the structural and functional diversification of thylakoidal membranes in land plants remains unclear. The *angulata10-1* (*anu10-1*) mutant, which exhibits pale-green rosettes, reduced growth, and deficient leaf lateral expansion, resulting in the presence of prominent marginal teeth, was isolated. Palisade cells in *anu10-1* are larger and less packed than in the wild type, giving rise to large intercellular spaces. The *ANU10* gene encodes a protein of unknown function that localizes to both chloroplasts and amyloplasts. In chloroplasts, ANU10 associates with thylakoidal membranes. Mutant *anu10-1* chloroplasts accumulate H_2_O_2_, and have reduced levels of chlorophyll and carotenoids. Moreover, these chloroplasts are small and abnormally shaped, thylakoidal membranes are less abundant, and their grana are absent due to impaired thylakoid stacking in the *anu10-1* mutant. Because the trimeric light-harvesting complex II (LHCII) has been reported to be required for thylakoid stacking, its levels were determined in *anu10-1* thylakoids and they were found to be reduced. Together, the data point to a requirement for ANU10 for chloroplast and mesophyll development.

## Summary

This study characterized ANU10, a novel chloroplast protein encoded by a nuclear gene in *Arabidopsis*, which influences leaf and chloroplast shape and is required for thylakoid stacking and grana formation.

## Introduction

In land plants, mutants with defective pigment content have allowed the identification of numerous nuclear genes that are crucial for organelle division and other plastid-specific processes (Lopez-Juez and Pyke, 2005; Hricová *et al.*, 2006). Many of these genes encode plant-specific proteins whose closest homologues are found in Cyanobacteria, as expected if they have been acquired by the nuclear genome in a horizontal transfer event, originating in the genome of the cyanobacterial ancestor of plastids. As an example, the nuclear genomes of land plants encode homologues of Filamenting temperature-sensitive Z (FtsZ) (Osteryoung and Vierling, 1995; Osteryoung *et al.*, 1998), Minicell D (MinD) (Colletti *et al.*, 2000), MinE (Itoh *et al.*, 2001), and many other components of the prokaryotic cell division apparatus.

The chloroplasts of land plants contain internal membrane systems, the thylakoids, which are arranged in stacks called grana. Grana thylakoids form cylindrical stacks that are connected to adjacent grana by non-stacked stroma thylakoids, which form right-handed helices around the grana (Austin and Staehelin, 2011). The thylakoidal membranes harbour the photosynthetic protein complexes, photosystems I and II (PSI and PSII), and other protein complexes, including ATP synthase, cytochrome *b*_6_ *f*, and the light-harvesting complexes I and II (LHCI and LHCII). Grana underlie a differential specialization of thylakoid membranes in the lateral dimension: the PSII and LHCII complexes are more abundant in grana, and the PSI and ATP synthase are preferentially found in the adjacent stroma thylakoids (Albertsson, 2001; Dekker and Boekema, 2005). Because grana have not been found in Cyanobacteria, the evolutionary origin of genes controlling the structural and functional diversification of thylakoidal membranes in land plants remains unclear (Mullineaux, 2005). Mutants with abnormal thylakoid stacking and loss of typical grana have long been known in species such as maize (Bachmann *et al.*, 1969), barley (Nielsen *et al.*, 1979), tobacco (Archer and Bonnett, 1987), and *Arabidopsis thaliana* (hereafter, *Arabidopsis*) (Reiter *et al.*, 1994). However, the molecular basis of the phenotypes observed in these species has only been determined in a few cases. Mutations in the *Arabidopsis CHLORATA-42* (*CH-42*) gene (Koncz *et al.*, 1990), which encodes one of the three subunits of the chloroplast magnesium chelatase complex, significantly reduce thylakoid stacking, suggesting a link between chlorophyll biosynthesis and grana formation (Apchelimov *et al.*, 2007). Another *Arabidopsis* gene, *GRANA-DEFICIENT CHLOROPLAST1* (*GDC1*), encodes an ankyrin-domain protein that is essential for grana formation. GDC1 is required for the assembly of the trimeric forms of the LHCII complex, which are barely detected in the *gdc1-3* mutant (Cui *et al.*, 2011). Current models emphasize the role of LHCII trimers in the formation of grana and the stacking of thylakoidal membranes (Mullet and Arntzen, 1980; Day *et al.*, 1984; Garab and Mustardy, 1999; Allen and Forsberg, 2001), mainly through electrostatic interactions between the positively charged N-terminal domains of LHCII trimers (stroma-exposed) and the negatively charged surface of LHCII trimers in adjacent thylakoidal membranes (Steinback *et al.*, 1979; Standfuss *et al.*, 2005). In addition, members of the CURVATURE THYLAKOID1 (CURT1) family of proteins have recently been shown to be enriched at the margin of grana, where they are thought to promote the curvature of thylakoidal membranes in *Arabidopsis*. Interestingly, the *Arabidopsis* CURT1A protein can replace the function of a distant cyanobacterial orthologue, showing that at least some proteins with crucial roles in thylakoid architecture are evolutionarily conserved, even though Cyanobacteria lack grana (Armbruster *et al.*, 2013).

Here it is shown that the *ANGULATA10* (*ANU10*) gene of *Arabidopsis* encodes a novel plastid-localized protein that is conserved among land plants, and the phenotypic and molecular characterization of loss-of-function *anu10* mutants is reported. It is demonstrated that the ANU10 protein localizes to plastids, including amyloplasts and chloroplasts, where it is associated with thylakoidal membranes. Thylakoid biogenesis is seriously impaired in *anu10-1* chloroplasts. Supporting the implication of LHCII trimers in grana formation, the *anu10-1* mutant contains reduced levels of LHCII trimers and shows defective thylakoid stacking, therefore lacking typical grana. A relationship between plastid integrity and leaf development can be inferred from the larger, sparsely packed cells observed in the palisade mesophyll of the *anu10-1* mutant.

## Materials and methods

### Plant material and growth conditions

*Arabidopsis thaliana* (L.) Heynh. wild-type accessions Columbia-0 (Col-0) and Landsberg *erecta* (L*er*), as well as the T-DNA insertion lines SAIL_708_F05 (N831342) and SAIL_659_F07 (N828696), were obtained from the Nottingham Arabidopsis Stock Centre (NASC). The *anu10-1* mutant was isolated after ethylmethane sulphonate (EMS)-induced mutagenesis as previously described (Berná *et al.*, 1999). All plants in this work were grown on Murashige and Skoog agar medium (2.15g l^–1^), at 20±1 °C and 60–70% relative humidity under continuous fluorescent light (~90 μmol m^−2^ s^−1^) as previously described (Ponce *et al.*, 1998). For the reactive oxygen species (ROS) production assay, low intensity (~55 μmol m^−2^ s^−1^) and moderately high intensity (~180 μmol m^−2^ s^−1^) light conditions were additionally used. Crosses and allelism tests were performed as reported in Berná *et al*. (1999).

### Positional cloning and molecular characterization of *anu10* alleles

Low-resolution mapping of the *anu10-1* mutation was performed as described in Ponce *et al.* (2006). For fine mapping, the nga392, SO392, and cer479911 insertion/deletion polymorphisms from Monsanto (http://www.arabidopsis.org/browse/Cereon) and the F3M18 and F1K23 single nucleotide polymorphisms (SNPs) from the 1001 genomes project database (Weigel and Mott, 2009) were used. To find the *anu10-1* mutation, a 3918bp fragment encompassing the entire transcription unit of At1g28530 was PCR amplified from L*er* and *anu10-1* genomic DNA, and sequenced on an ABI PRISM 3130xl Genetic Analyser (Applied Biosystems). All the primers used for the cloning and sequencing of At1g28530 are listed in Supplementary Table S1 available at *JXB* online. The T-DNA insertions in N831342 and N828696 lines were confirmed by PCR using primers recommended by the ‘T-DNA Primer Design’ tool (http://signal.salk.edu/tdnaprimers.2.html; Supplementary Table S1).

### Bioinformatic analyses

Four cDNA (BT005784.1, BT008607.1, BX816836.1, and AK228671.1) and nine expressed sequence tag (EST; AU228187.1, AU237163.1, BP599159.1, BP606540.1, BP662266.1, BP807381.1, BP846942.1, ES017536.1, and EL984216.1) sequences from GenBank were used to assemble the transcriptional unit of At1g28530 using CAP3 (http://pbil.univ-lyon1.fr/cap3.php) (Huang and Madan, 1999). Subcellular localization was predicted with TargetP (http://www.cbs.dtu.dk/services/TargetP/) (Emanuelsson *et al.*, 2000) and Multiloc2 (http://abi.inf.uni-tuebingen.de/Services/MultiLoc2) (Blum *et al.*, 2009). Chloroplast transit peptide sequences and transmembrane domains were predicted with ChloroP 1.1 (http://www.cbs.dtu.dk/services/ChloroP/) (Emanuelsson *et al.*, 1999) and SOSUI (http://bp.nuap.nagoya-u.ac.jp/sosui/) (Hirokawa *et al.*, 1998), respectively.

To identify ANU10 homologues, BLASTP searches (Altschul *et al.*, 1997) were carried out at the NCBI server using a word size of 2 and default values for all other parameters. Full-length sequences were selected based on a BLAST E-value cut-off of 3×10^–6^, and were subsequently aligned using the consistency-based method implemented in T-Coffee (Notredame *et al.*, 2000). Alignments were refined with MUSCLE (http://www.ebi.ac.uk/Tools/msa/muscle/) (Edgar, 2004) and shaded with BOXSHADE3.21 (http://www.ch.embnet.org/software/BOX_form.html). Identity percentages were calculated using the Sequence Manipulation Suite (http://www.bioinformatics.org/sms2/index.html) (Stothard, 2000). Phylogenetic trees were obtained using MEGA5 (Tamura *et al.*, 2011). Searches for distant homologues of ANU10 were carried out with HMMER (http://hmmer.janelia.org/) (Finn *et al.*, 2011).

### RNA isolation, cDNA synthesis, and qRT-PCR

Total RNA from Col-0 rosettes [collected 21 d after stratification (das)] was extracted using TRI Reagent (Sigma), and DNA was removed using the TURBO DNA-free Kit (Invitrogen). First-strand cDNA was synthesized using random hexamers and the Maxima Reverse Transcriptase system (Fermentas). For quantification of the expression of nuclear and plastid genes, the primers listed on Supplementary Table S1 available at *JXB* online were used. The 18S rRNA gene was used as an internal control (Yamauchi *et al.*, 2004). Three different biological replicates and triplicate reactions were used. Amplification reactions were prepared in a volume of 20 μl by adding 7.5 μl of Maxima SYBR Green/ROX qPCR Master Mix (Fermentas), 3 μl of the corresponding primer pair (2.5 μM each), and 1 μl of cDNA template. Relative quantification of gene expression data was performed using the comparative *C*_T_ method (Schmittgen and Livak, 2008) on a Step One Plus System (Applied Biosystems).

### Gene constructs and plant transformation

To make the *35S*_*pro*_ *:ANU10* and *35S*_*pro*_ *:ANU10:GFP* (green fluorescent protein) transgenes, the full-length coding sequence of *ANU10* was amplified from Col-0 cDNA using Phusion polymerase (Thermo Scientific) with the ANU10cds-F and ANU10cds-R primers (Supplementary Table S1 at *JXB* online). The amplification product was cloned into the pENTR/D-TOPO entry vector (Invitrogen) and transferred into the pMDC32 and pMDC83 destination vectors, which include a dual 35S promoter (Curtis and Grossniklaus, 2003). For the *ANU10*_*pro*_ *:GUS* (β-glucuronidase) transgene, a 1.5-kb fragment encompassing the intergenic region between At1g28530 and At1g28540, including the At1g28530 5′-untranslated region (UTR) and At1g28540 3′-UTR, was amplified using Col-0 genomic DNA as the template. The resulting product was cloned into the pGEM-T Easy221 vector (kindly provided by B. Scheres), and then transferred into the pMDC163 destination vector (Curtis and Grossniklaus, 2003). Alternatively, the *ANU10* promoter region was fused with the ANU10 full-length cDNA in a two-template PCR of overlapping products, using the primers ANU10cds-pro and ANU10pro-cds (Supplementary Table S1). The resulting fusion product was cloned into the pGEM-T Easy221 vector and transferred into pMDC111 (Curtis and Grossniklaus, 2003) to obtain the *ANU10*_*pro*_ *:ANU10:GFP* construct. All the constructs were transformed into *Agrobacterium tumefaciens* LBA4404. Col-0, L*er*, and *anu10-1* plants were transformed by the floral dip method (Clough and Bent, 1998). T_1_ transgenic plants were selected on plates supplemented with 15 μg ml^–1^ hygromycin B (Invitrogen).

### Microscopy, histology, and morphometry

For transmission electron microscopy, mutant, wild-type, and transgenic plants were harvested 16 das. Leaf tissue excluding the primary vein and leaf margin was excised and fixed in McDowell’s solution (McDowell and Trump, 1976), and prepared as previously described (Hricová *et al.*, 2006). Samples were visualized at 80kV using a JEOL 1011 transmission electron microscope equipped with a Gatan 792 BioScan digital camera. Grana diameter and height measurements were obtained from transmission electron micrographs using the ImageJ software. Leaf tissues were imaged using a Leica TCS SPE confocal microscope. Root tissues were imaged using a Nikon C1 confocal microscope. Transverse sections of leaves were obtained as described by Serrano-Cartagena *et al.* (2000), embedding the tissue in Technovit7100 resin and obtaining 10 μm sections. Rosette area measurement and morphometric analysis of palisade cells and transverse sections of leaves were performed as described previously (Pérez-Pérez *et al.*, 2011; Ferrández-Ayela *et al.*, 2013).

### Dry weight and pigment determination

For dry weight measurement, eight plants of each genotype were oven-dried for 48h at 55 ºC. For determination of chlorophylls and carotenoids, four independent samples of 100mg of fresh leaves from rosettes collected 16 das were pooled, frozen in liquid N_2_, and homogenized with 3.5ml of cold 80% acetone. The samples were centrifuged for 5min at 5000rpm and the pigment concentration in the supernatant was spectrophotometrically determined as previously described (Wellburn, 1994).

### ROS, GUS, and Lugol staining

To determine ROS accumulation, a minimum of six first-node leaves from each genotype were excised and incubated overnight in 1mg ml^–1^ 3,3′-diaminobenzidine (DAB; Sigma-Aldrich), under vacuum and in the dark. Leaves were cleared by boiling in acetic acid:glycerol:ethanol (1:1:3 v/v/v) for 5min, and then incubated in 96% (v/v) ethanol until chlorophyll was bleached. GUS staining of plant tissues and Lugol staining of root tips were performed as described in Robles *et al.* (2010) and Willemsen *et al.* (1998), respectively. Samples were visualized with a Nikon C1 microscope.

### Western blot analysis of chloroplast proteins and blue native PAGE of thylakoid membranes

Chloroplasts from rosette leaves collected 16 das were isolated as described by Grabsztunowicz and Jackowski (2012), omitting the Percoll step gradient. Stroma and thylakoid membranes from isolated chloroplasts were separated as previously reported (Armbruster *et al.*, 2010). A volume of isolated chloroplasts and thylakoids equivalent to 20 μg of chlorophyll, as well as 20 μg of proteins from the stroma fraction, including a lane with an EZ-Run Pre-stained *Rec* Protein Ladder (Fisher BioReagents), were resolved by 10% SDS–PAGE, blotted on nitrocellulose membranes (Amersham Hybond ECL, RPN203D; GE Healthcare), and subjected to immunoblot analysis with specific antibodies (G1544, Sigma; AS03 037-10 and AS05 092, Agrisera AB). Isolation of thylakoids from rosettes collected 16 das and blue native PAGE were carried out as reported in Pérez-Pérez *et al*. (2013).

## Results

### Leaf and whole-plant defects in *anu10-1* plants

In a large-scale screen for EMS-induced mutants with abnormal leaf shape, 18 mutants with pale-green leaves and dentate margins were previously identified. The causative mutations were dubbed *angulata* (*anu*), and complementation tests showed that they damage 12 different genes (*ANU1*–*ANU12*) (Berná *et al.*, 1999).

**Fig. 1.**
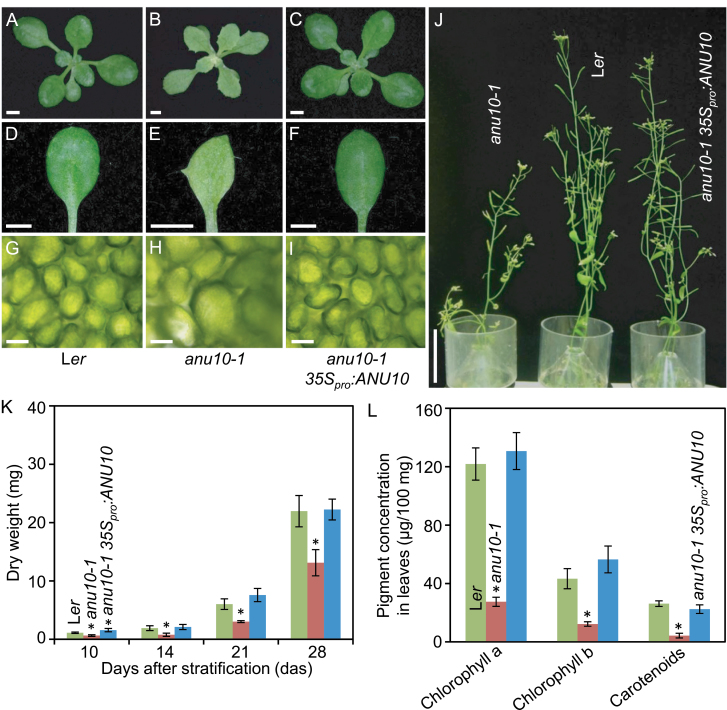
Phenotypic characterization and rescue of the *anu10-1* mutant. (A–C) Rosettes, (D–F) first-node leaves, and (G–I) bright-field micrographs of the subepidermal layer of palisade mesophyll cells from (A, D, G) the L*er* wild type, (B, E, H) the *anu10-1* mutant, and (C, F, I) a transgenic *anu10-1 35Spro:ANU10* plant. (J) Adult plants. Pictures were taken (A–I) 16 and (J) 42 das. Scale bars indicate (A–F) 2mm, (G–I) 30 μm, and (J) 5cm. (K) Dry weight and (L) pigment content in L*er*, *anu10-1*, and transgenic *anu10-1 35Spro:ANU10* plants. Error bars indicate standard deviations. Asterisks indicate values significantly different from L*er* in a Mann–Whitney U-test [(K) *P*<0.01, *n*=8 and (L) *P*<0.05, *n*=4].

**Fig. 2.**
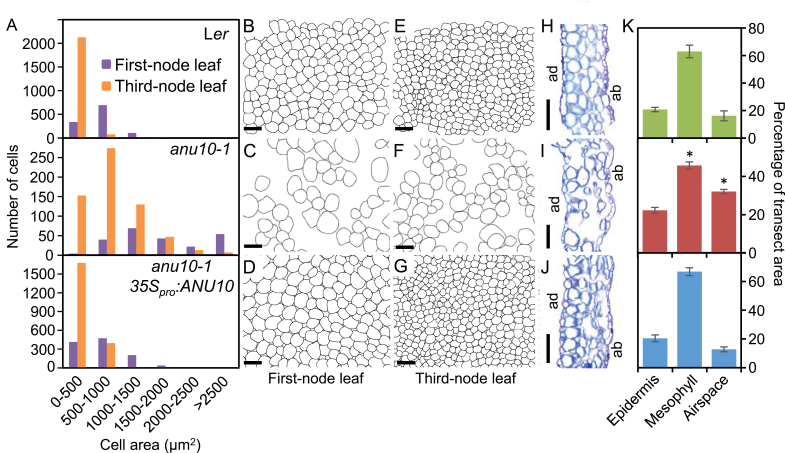
Morphometry of *anu10-1* mesophyll cells. (A) Distribution of palisade mesophyll cell area in first- and third-node leaves from L*er*, *anu10-1*, and *anu10-1 35S*_*pro*_ *:ANU10* plants (*n*=8). (B–G) Representative diagrams of the subepidermal layer of palisade mesophyll cells from (B–D) first- and (E–G) third-node leaves. Diagrams were drawn from differential interference contrast pictures taken from cleared leaves. (H–J) First-node leaf transverse sections from (H) L*er*, (I) *anu10-1*, and (J) *anu10-1 35S*_*pro*_ *:ANU10* plants. ad, adaxial surface, ab, abaxial surface. Scale bars indicate (B–G) 50 μm and (H–J) 500 μm. (K) Percentage of leaf transect area occupied by epidermis, mesophyll (including palisade and spongy mesophyll cells and bundle sheath cells), or air spaces in L*er* (green), *anu10-1* (red), and *anu10-1 35S*_*pro*_ *:ANU10* (blue) first-node leaves. Error bars indicate standard deviations. Asterisks indicate values significantly different from L*er* in a Mann–Whitney U-test (*P*<0.01, *n*=6).

The only mutant allele of the *ANU10* gene, *anu10-1*, causes reduced rosette and stem growth (Fig. 1A, B, J). The dry weight and projected area of *anu10-1* rosettes were significantly lower than in wild-type L*er* plants throughout the study (Fig. 1K; Supplementary Fig. S1 at *JXB* online). Rosettes of the *anu10-1* mutant harvested 21 das showed a dry weight of 3.02±0.16mg and an area of 147.72±32.68mm^2^, while the corresponding values for L*er* were 6.02±0.91mg and 701.48±56.88mm^2^, respectively. Adult *anu10-1* plants also exhibited a significant reduction in shoot length: 28.47±2.69cm in L*er*, but only 18.22±3.55cm in *anu10-1* 42 das (Fig. 1J; Supplementary Fig. S1). The number of secondary stems was lower in *anu10-1* (4.67±0.52) than in L*er* (9.25±0.71) determined 35 das, although this difference disappeared over time (Supplementary Fig. S1). The leaves of *anu10-1* were pale green and their margins had prominent teeth (Fig. 1E). As seen in paradermal sections, the palisade mesophyll cells were irregular in size, with larger cells in *anu10-1* than in the wild type (Fig. 1G, H). By measuring the size of individual cells, a shift in the distribution of palisade cell size towards greater values (Fig. 2A), as well as the presence of large intercellular spaces in the palisade mesophyll of *anu10-1* leaves (Fig. 2B, C, E, F) was detected. Similar defects were observed in transverse sections of first-node leaves (Fig. 2H, I). In these sections, mesophyll cells filled a significantly smaller percentage of the section area, which was matched by a significant increase in the area occupied by air spaces in *anu10-1* leaves (Fig. 2K).

To gain insight into the physiological basis of the pale-green phenotype of *anu10-1*, pigment levels were measured in the mutant. In line with the observed pale-green phenotype (Fig. 1B, E), a reduction in the levels of chlorophyll *a*, chlorophyll *b*, and carotenoids was detected (Fig. 1L). Because carotenoids play an important role in photoprotection, the presence of ROS, visualized by staining with DAB, was also tested. Darker staining was observed in *anu10-1* leaves relative to L*er* (Supplementary Fig. S2 available at JXB online), which indicates the presence of increased levels of H_2_O_2_. In the mutant, the accumulation of H_2_O_2_ was positively correlated with light intensity (Supplementary Fig. S2).

### Positional cloning of *ANU10*

**Fig. 3.**
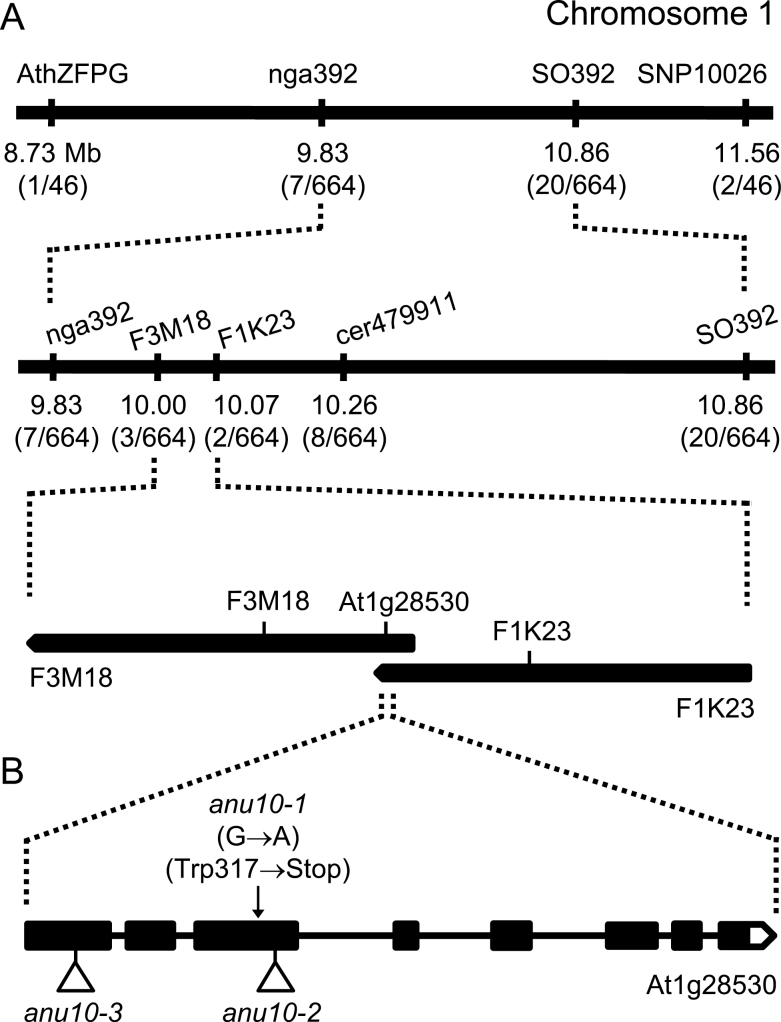
Positional cloning of *ANU10*. (A) A mapping population of 332 F_2_ plants derived from an *anu10-1×*Col-0 cross allowed a candidate region of 72kb to be defined in chromosome 1. Names and physical map positions of the molecular markers used for linkage analysis are shown. The number of recombinant chromosomes found and the total number of chromosomes analysed are indicated in parentheses. (B) Structure of the *ANU10* gene with indication of the nature and position of the *anu10* mutations. Boxes and lines indicate exons and introns, respectively. A white box represents the 3′-UTR. Triangles indicate T-DNA insertions.

The *anu10-1* mutation was previously mapped to chromosome 1 (Robles and Micol, 2001). To understand further the molecular basis of the phenotype of *anu10-1*, a positional cloning approach was undertaken to identify the causal gene. First a 72kb candidate interval (Fig. 3A) flanked by two SNP markers, F3M18 and F1K23 (Supplementary Table S1 available at *JXB* online), was defined. This interval encompassed 24 candidate genes. Because the phenotype of *anu10-1* suggests a defect in a chloroplast-related function (Fig. 1B, E), the At1g28530 gene, which is the only gene in this interval predicted to encode a chloroplast-localized protein, was the focus of further study. The At1g28530 gene was sequenced in mutant and wild-type plants, and a G→A transition was found in its coding region only in *anu10-1* mutant plants (Fig. 3B). Expression of the At1g28530 gene is supported by four cDNA and nine EST sequences deposited in GenBank (see the Materials and methods). In order to determine the intron–exon structure of the At1g28530 gene, these sequences were assembled using CAP3 and the consensus mRNA sequence was aligned to the sequence of *Arabidopsis* chromosome 1 (NC_003070). The consensus mRNA sequence has eight exons and its longest open reading frame encodes a protein with 614 amino acids and a molecular mass of 68.46kDa. The *anu10-1* mutation introduces a stop codon (Trp317→Stop) in the third exon of the gene (Fig. 3B), and is predicted to truncate the protein prematurely (from 614 amino acids in L*er* to only 316 in *anu10-1*).

To identify additional alleles of the *ANU10* gene, two publicly available lines, SAIL_708_F05 and SAIL_659_F07, which carry T-DNA insertions in the coding sequence of At1g28530 were characterized. When homozygous for the T-DNA insertion, these two lines displayed phenotypes similar to those of *anu10-1*, including leaves with prominent marginal teeth and paler rosettes, when compared with their wild-type Col-0 (Supplementary Fig. S3A–D at *JXB* online). In general, these phenotypes were less severe than those of *anu10-1* and were more apparent in younger rosettes, becoming indistinguishable from Col-0 14–15 das. The mutant phenotype of the F_1_ progeny from the crosses of *anu10-1* to SAIL_708_F05 and SAIL_659_F07 indicated that both T-DNA lines carry *anu10* alleles (Supplementary Fig. S3E, F), which were named *anu10-2* and *anu10-3* (Fig. 3B). Because the *anu10-2* and *anu10-3* mutants carry T-DNA insertions in exons 3 and 1, respectively, which are expected to disrupt the coding potential of the mRNA, it is speculated that the milder effects of these mutant alleles might reflect differences between the L*er* and Col-0 genetic backgrounds.

To confirm further the identity of the *ANU10* gene, a construct was made to express the coding region of At1g28530 constitutively. When transformed into *anu10-1* plants, the *35S*_*pro*_ *:ANU10* transgene fully complemented the defects in leaf shape (Fig. 1C, F), growth (Fig. 1C, J; Supplementary Fig. S1 at *JXB* online), mesophyll development (Figs 1I, 2A, D, G, J, K), pigment levels (Fig. 1L), and H_2_O_2_ accumulation (Supplementary Fig. S2) in each of six independent transformants. Therefore, the correct identification of *ANU10* as the At1g28530 gene is supported both by the transgenic complementation studies and by the independent isolation of three mutant alleles carrying lesions in the coding region of At1g28530.

### Analysis of *ANU10* expression

**Fig. 4.**
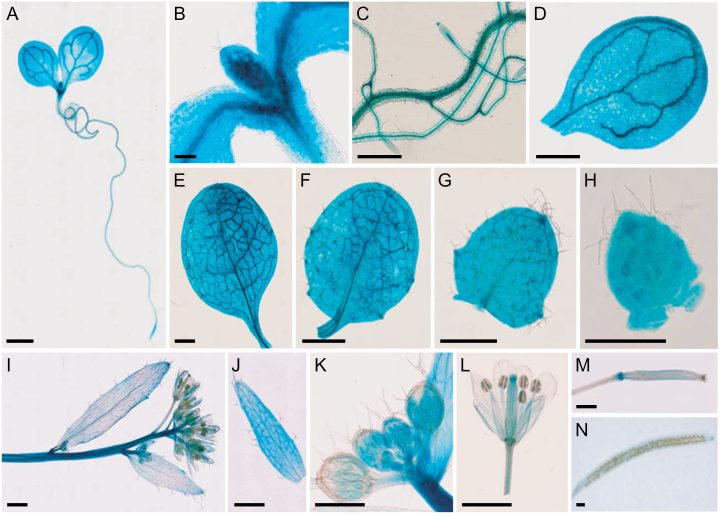
Visualization of *ANU10*_*pro*_ *:GUS* activity in a wild-type background. (A) Seedling, (B) detail of the shoot apex, (C) roots, and (D) cotyledon. (E–H) Vegetative leaves from the (E) first, (F) third, (G) fifth, and (H) seventh nodes. (I) Inflorescence, (J) young cauline leaf, (K) immature flowers, (L) mature flower, and (M) immature and (N) mature siliques. Pictures were taken (A) 3, (B) 6, (C–H) 13, and (I–N) 42 das. Scale bars indicate (A, D–G, I, J, L–N) 1mm, (C, H, K) 500 μm, and (B) 100 μm.

To determine the expression pattern of *ANU10* in wild-type plants, an *ANU10*_*pro*_ *:GUS* reporter transgene was constructed. Four independent transgenic plants stably expressing the transgene and showing the same staining pattern were studied. The GUS signal was broad in seedlings collected 3 das (Fig. 4A), being particularly intense in incipient leaves (Fig. 4B). In plants collected 13 das, GUS activity was detected in roots, cotyledons, and leaves (Fig. 4C–H). In older plants, GUS activity was observed in stems, cauline leaves, flowers, and siliques (Fig. 4I–N). Remarkably, the GUS signal was more intense in young cauline leaves (Fig. 4J), flowers (Fig. 4K), and siliques (Fig. 4M) than in the corresponding mature organs (Fig. 4I, L, N). The spatio-temporal expression analysis suggests that *ANU10* is expressed in all plant organs and that its expression is particularly important in developing organs.

### The *ANU10* gene encodes a protein of unknown function

*ANU10* is a single-copy gene in the nuclear genome of *Arabidopsis*. Because the gene is predicted to encode a protein of unknown function with no conserved domains, BLAST searches were carried out to identify similar protein sequences in public databases. Significant hits were found in the genomes of other higher plants and the moss *Physcomitrella patens*, but not in those of animals or other eukaryotes, including algae, suggesting that ANU10 belongs to a family of embryophyte-specific proteins (Supplementary Fig. S4 available at *JXB* online). The chloroplast localization of ANU10 was consistently predicted by several computational tools (see the Materials and methods), including TargetP (score=0.929) and Multiloc2 (score=0.57). ChloroP 1.1 predicted a chloroplast transit peptide in ANU10 and in most of its orthologues from other land plants (Supplementary Fig. S4A, Supplementary Table S2). In addition to the transit peptide, ANU10 and most of its orthologues were also predicted to have a transmembrane domain (Supplementary Fig. S4A, Supplementary Table S2), suggesting that these proteins are anchored to chloroplast membranes. As an example, SOSUI predicted a transmembrane domain spanning residues 421–443 in ANU10.

To gain insight into the evolutionary origin of this protein family, HMMER searches were carried out using a profile made with the sequences of several ANU10 homologues from land plants. HMMER allowed the identification of some distantly related sequences in Cyanobacteria. In line with these results, a search for known domains in the Pfam database (Punta *et al.*, 2012) yielded a low significance hit to a domain of unknown function (DUF4335) present in some cyanobacterial proteins. Together, these data indicate that ANU10 is conserved among land plants. However, unlike proteins such as CURT1A (Armbruster *et al.*, 2013), which is functionally conserved in Cyanobacteria, the search for cyanobacterial orthologues of ANU10 did not yield obvious candidates.

### ANU10 localizes to plastids

**Fig. 5.**
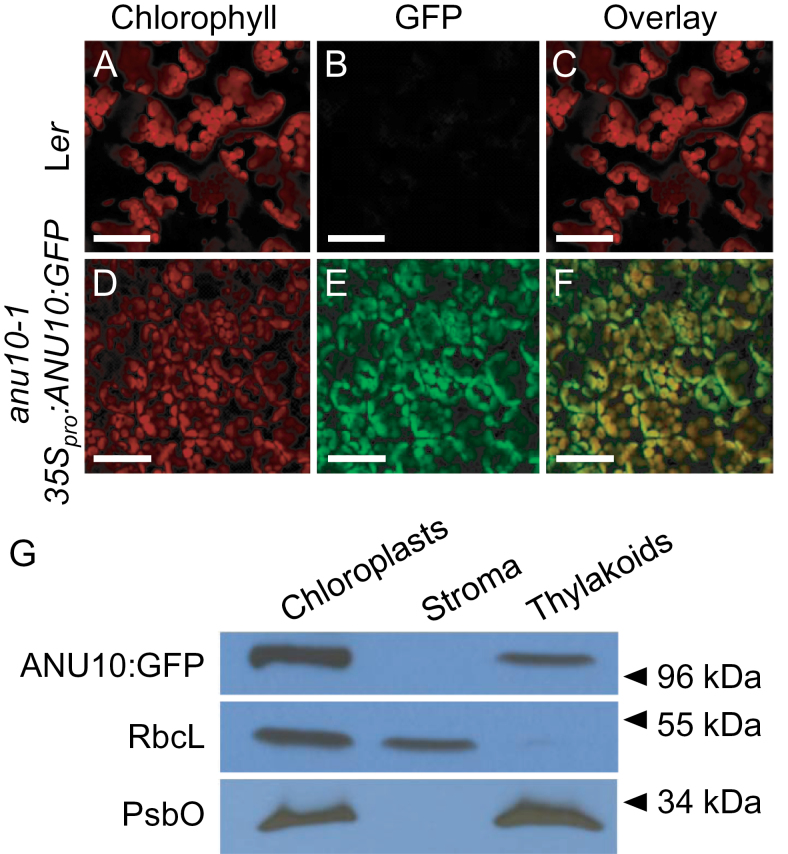
Subcellular and suborganellar localization of ANU10. (A–F) Confocal micrographs of the subepidermal layer of palisade mesophyll cells from (A–C) L*er* and (D–F) *anu10-1 35S*_*pro*_ *:ANU10:GFP* transgenic plants. Micrographs show (A, D) the chlorophyll autofluorescence of the chloroplasts, (B, E) the GFP fluorescence, and (C, F) an overlay of the chlorophyll and GFP signals, showing their co-localization in (F). Pictures were taken from first-node leaves collected 16 das. Scale bars indicate 50 μm. (G) Western blot analysis of the proteins in chloroplast, stroma, and thylakoid fractions isolated from *anu10-1 35S*_*pro*_ *:ANU10:GFP* transgenic plants collected 16 das. Primary antibodies against GFP, the large Rubisco subunit (RbcL), and the PsbO subunit of PSII were used. Molecular mass markers are indicated on the right.

To determine experimentally the subcellular localization of the ANU10 protein, an in-frame translational fusion of GFP to the C-terminal end of ANU10 was made. Transgenic *anu10-1* plants expressing *35S*_*pro*_ *:ANU10:GFP* were used to visualize the GFP signal by confocal laser scanning microscopy. The GFP signal was specifically detected in chloroplasts from four independent lines carrying the *35S*_*pro*_ *:ANU10:GFP* transgene (Fig. 5A–F). To exclude that the observed localization pattern represents an artefact due to overexpression of the ANU10:GFP fusion protein, the same translational fusion was also placed under the control of the endogenous *ANU10* promoter (*ANU10*_*pro*_ *:ANU10:GFP*). An identical distribution of GFP signal was observed in *anu10-1* plants expressing the *ANU10*_*pro*_ *:ANU10:GFP* transgene (Supplementary Fig. S5 at *JXB* online), demonstrating that the promoter chosen does not affect the subcellular localization of the fusion protein. Interestingly, both transgenes were able partially to rescue the *anu10-1* phenotype in young rosettes (Supplementary Fig. S6). The rescue was complete 21 das regardless of the promoter used, indicating that the ANU10:GFP fusion protein retains sufficient activity to complement the mutant phenotype at this stage. To determine the suborganellar localization of the ANU10 protein, stromal and thylakoidal fractions were isolated from the chloroplasts of *anu10-1 35S*_*pro*_ *:ANU10:GFP* transgenic plants. The presence of the ANU10:GFP fusion protein in each fraction was tested by western blotting using an anti-GFP antibody. The fusion protein was specifically detected in thylakoids, but not in the soluble stromal fraction (Fig. 5G), indicating that ANU10 is associated with thylakoidal membranes.

**Fig. 6.**
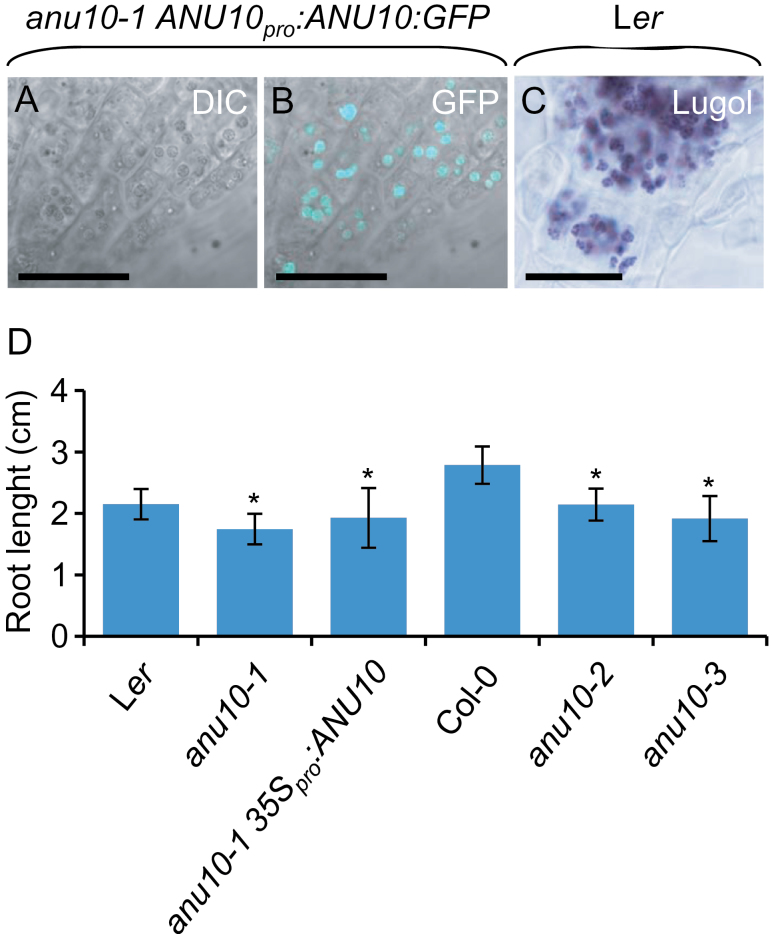
Localization of the ANU10 protein and effect of *anu10* mutations on root growth. (A, B) Confocal micrographs of the root apex from an *anu10-1 ANU10*_*pro*_ *:ANU10:GFP* transgenic plant: (A) differential interference contrast (DIC) image and (B) an overlay of the GFP fluorescence and the DIC image. (C) Root apex from a L*er* plant stained with lugol. Scale bars indicate 30 μm. (D) Main root length of *anu10-1*, *anu10-2*, and *anu10-3* mutants, their respective wild types, and *anu10-1 35S*_*pro*_ *:ANU10* transgenic plants. Error bars indicate standard deviations. Asterisks indicate values significantly different from the wild type in a *t*-test (*P*<0.05, *n*=30).

Because *ANU10* was found to be expressed in roots (Fig. 4C), root tissues were also examined under a confocal microscope. A strong GFP signal was detected in root tips from four independent lines expressing the *ANU10*_*pro*_ *:ANU10:GFP* transgene (Fig. 6A, B). The pattern of GFP signal paralleled the distribution of amyloplasts in root tips stained with lugol (Fig. 6C). Although amyloplasts play a central role in root graviperception, no obvious defects were detected in the gravitropic response of *anu10* roots (Supplementary Fig. S7A at *JXB* online). Starch content was similar between *anu10* mutants and the wild type, as visualized after lugol staining (Supplementary Fig. S7B). The primary root length was reduced in *anu10-1* (1.75±0.25cm) compared with L*er* (2.15±0.25cm) (Fig. 6D). Roots of *anu10-2* and *anu10-3* were also shorter (2.14±0.26 and 1.92±0.37cm, respectively) than those of Col-0 (2.79±0.30cm). However, this phenotype, unlike the leaf phenotypes, was not restored *in anu10-1 35S*_*pro*_ *:ANU10* transgenic plants, in which the mean length of the main root was 1.93±0.49cm (Fig. 6D).

### Thylakoid membranes and LHCII trimers are less abundant in *anu10-1*

**Fig. 7.**
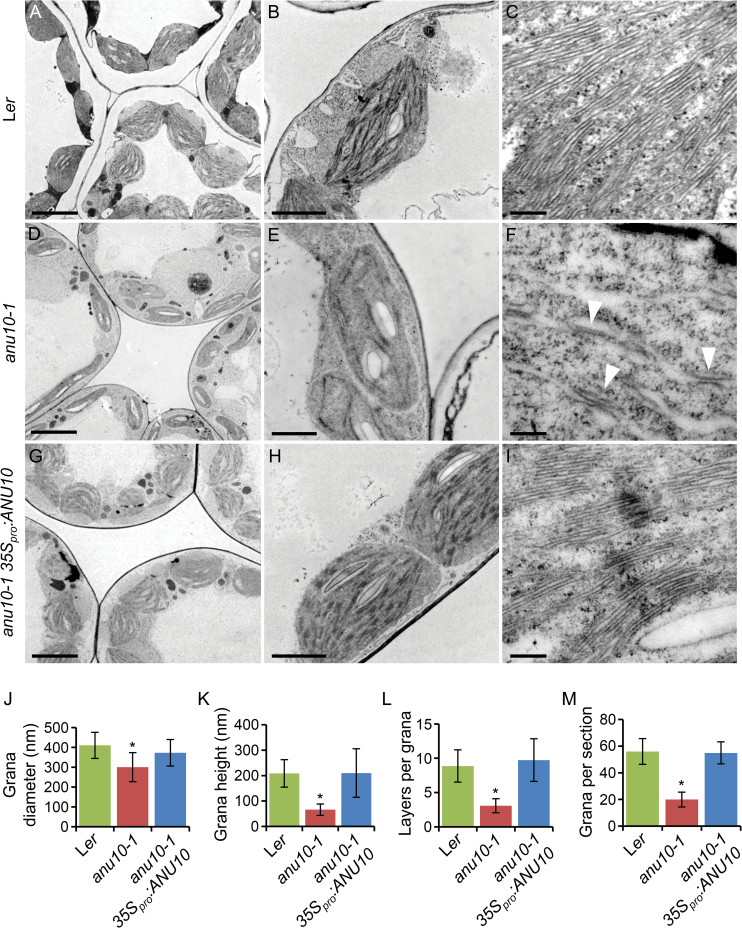
Ultrastructure of *anu10-1* chloroplasts. Transmission electron micrographs of palisade mesophyll cell chloroplasts from (A–C) L*er*, (D–F) *anu10-1*, and (G–I) *anu10-1 35S*_*pro*_ *:ANU10*. Arrowheads in (F) indicate unstacked thylakoid membranes in the *anu10-1* mutant. Pictures were taken from first-node leaves collected 16 das. Scale bars indicate (A, D, G) 5 μm, (B, H) 2 μm, (E) 1 μm, and (C, F, I) 200nm. (J–M) Comparison of (J) the diameter (*x*-axis) and (K) height (*y*-axis) of granal stacks, (L) the number of membrane layers in granal stacks, and (M) the number of stacks in chloroplast sections from L*er*, *anu10-1*, and *anu10-1 35S*_*pro*_ *:ANU10*. The number of grana per chloroplast section was determined from images of individual chroroplasts similar to those shown in B, E, and H. Asterisks indicate values significantly different from the wild type in a *t*-test (*P*<0.05, *n*=10–20).

**Fig. 8.**
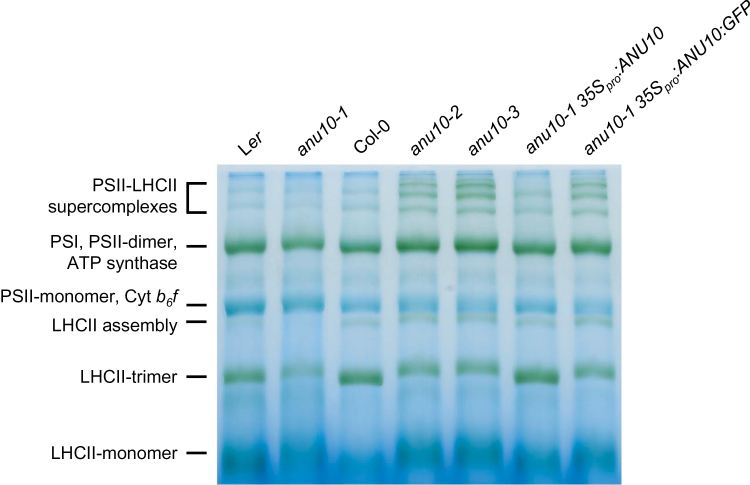
Thylakoidal protein complexes in the *anu10* mutants. Blue native PAGE of photosynthetic protein complexes from *anu10-1*, *anu10-2*, and *anu10-3* mutants, their respective wild types, and *anu10-1 35S*_*pro*_ *:ANU10* and *anu10-1 35S*_*pro*_ *:ANU10:GFP* transgenic plants. PSII, photosystem II; PSI, photosystem I; Cyt *b*_6_ *f*, cytochrome *b*_6_ *f* complex; LHCII, light-harvesting chlorophyll *a*/*b*-binding protein complex II.

To investigate whether ANU10 deficiency affects the suborganellar organization of chloroplasts, the ultrastructure of chloroplasts from L*er*, *anu10-1*, and *anu10-1 35S*_*pro*_ *:ANU10* mesophyll cells was studied using transmission electron microscopy. Chloroplasts were smaller in *anu10-1* plants than in the wild type. While L*er* chloroplasts are typically lens shaped (Fig. 7A, B), *anu10-1* chloroplasts were smaller and abnormally shaped (Fig. 7D, E). Fewer thylakoidal membranes were observed in *anu10-1* chloroplasts (Fig. 7B, E). When observed at high magnification, it was found that *anu10-1* thylakoids failed to stack and form typical grana. Under the conditions used here, wild-type grana were composed of 8.37±2.36 layers of thylakoidal membranes, while the number of layers per grana was only 3.08±1.02 for *anu10-1* (Fig. 7F, L), as also reflected by the decreased height of *anu10-1* grana (Fig. 7K). Moreover, the diameter of the grana and the number of grana per chloroplast section were found to be reduced in *anu10-1* (Fig. 7J, M). Chloroplast size, morphology, thylakoid abundance, and grana morphology and stacking were totally restored to those of the wild type in *anu10-1* plants expressing the *35S*_*pro*_ *:ANU10* transgene (Fig. 7G–I, J–M). Because LHCII trimers are thought to participate in thylakoid stacking, blue native PAGE was used to study the levels of trimeric LHCII complexes in isolated thylakoids from 16 das plants. LHCII trimers migrated slightly more slowly and their levels were reduced in thylakoids of *anu10-1* compared with those of L*er* (Fig. 8A, lanes 1 and 2). The *anu10-2* and *anu10-3* mutants showed a similar reduction in the amount and mobility of LHCII trimers relative to the Col-0 wild type (Fig. 8A, lanes 3–5). The mobility and levels of LHCII trimers were fully restored in thylakoids of *anu10-1* plants carrying the *35S*_*pro*_ *:ANU10* transgene (Fig. 8A, lane 6). The levels of LHCII trimers were partially restored to wild-type levels in *anu10-1 35S*_*pro*_ *:ANU10:GFP* plants (Fig. 8A, lane 7), in line with the partial phenotypic rescue seen in *anu10-1 35S*_*pro*_ *:ANU10:GFP* plants collected 16 das (Supplementary Fig. S6B at *JXB* online). Taken together, these results suggest that ANU10 is important for thylakoid biogenesis and grana formation, and do not allow the exclusion that a defect in the levels or composition of the LHCII trimers might be responsible for the reduced thylakoid stacking seen in the *anu10-1* mutant.

### Expression of nuclear and chloroplast genes in *anu10-1*

**Fig. 9.**
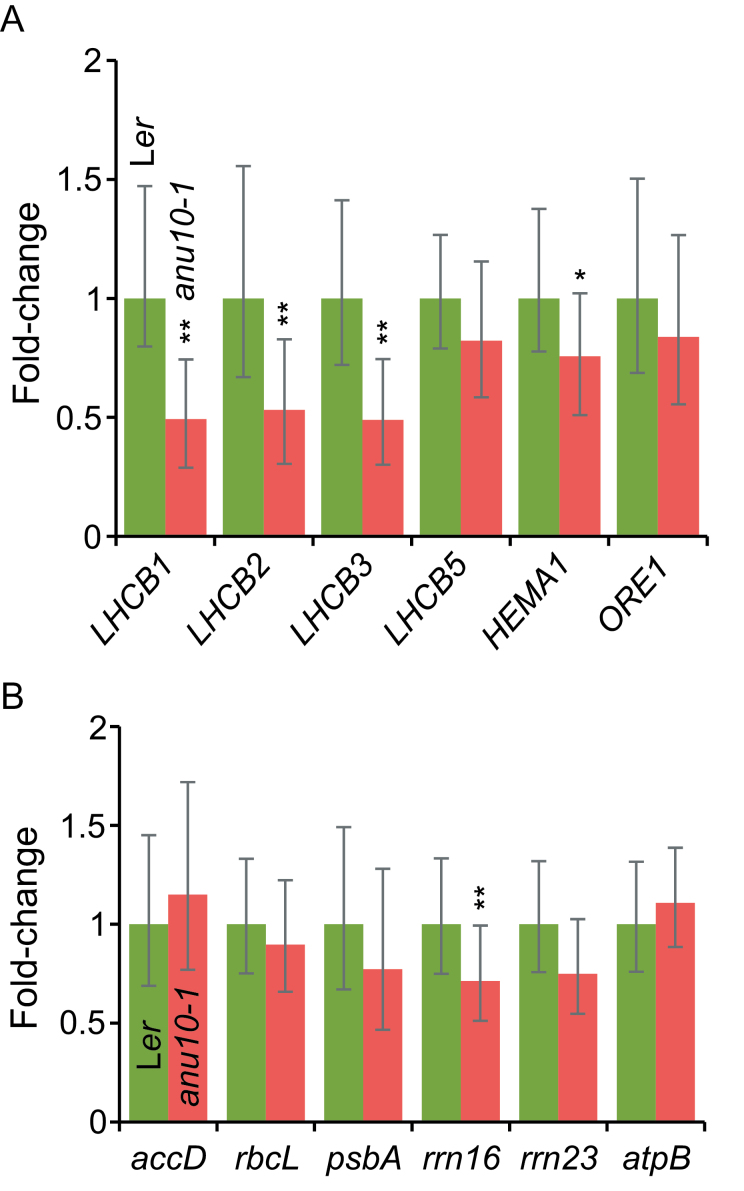
Expression of nuclear and plastid genes in the *anu10-1* mutant. (A, B) qRT-PCR analysis of the expression of (A) *LHCB1*, *LHCB2*, *LHCB3*, *LHCB5*, *HEMA1*, and *ORE1* nuclear genes, and (B) *accD*, *psbA*, *rbcL*, *rrn16*, *rrn23*, and *atpB* plastid genes in L*er* and *anu10-1* rosettes collected 16 das. Bars indicate the relative expression levels, determined by the comparative C_T_ method, and normalized with the expression of the 18S rRNA housekeeping gene. Error bars indicate the interval delimited by 2^–(ΔΔCT± SD)^. Asterisks indicate ΔC_T_ values significantly different from those of L*er* in a Mann–Whitney U-test (*P*<0.01; *n*=9).

Quantitative RT-PCR (qRT-PCR) was used to explore whether ANU10 deficiency affects the expression of nuclear and plastid genes. The relative expression of nuclear genes encoding subunits of LHCII trimers (*LHCB1*, *LHCB2* and *LHCB3*) was found to be ~0.5-fold decreased in *anu10-1* compared with L*er* (Fig. 9A). Because the *LHCB5* gene is known to be overexpressed in plants that do not express the LHCB1 and LHCB2 proteins (Ruban *et al.*, 2003), the expression of *LHCB5* was also analysed. However, the expression levels of *LHCB5* were not significantly different in *anu10-1* and L*er* (Fig. 9A). Because the impact of the *anu10-1* mutation on the expression of *LHCB* genes might be an indirect consequence of a more general problem that triggers retrograde signals from the chloroplast to the nucleus, the expression of the *HEMA1* gene, which is involved in chlorophyll biosynthesis and is particularly tightly regulated by chloroplast-to-nucleus signals (Mochizuki *et al.*, 2008), was also examined. The expression of *HEMA1* was significantly although mildly reduced in *anu10-1* (Fig. 9A), suggesting that the reduced expression of *LHCB* genes is at least in part an indirect effect of the mutation on these signalling pathways.

Because the phenotype of *anu10-1* mutants might also be interpreted as a consequence of premature senescence, which might lead to grana disassembly and thylakoid swelling (Evans *et al.*, 2010; Krupinska *et al.*, 2012), the expression level of the nucleus-encoded *ORESARA1* (*ORE1*) transcription factor, a senescence marker that has recently been reported to be a key regulator of the senescence response (Kim *et al.*, 2009; Balazadeh *et al.*, 2010), was also monitored. *ORE1* was expressed at similar levels in *anu10-1* mutants and wild-type rosettes (Fig. 9A), suggesting that premature senescence is not the cause of the observed phenotype.

To survey the effects of the *anu10-1* mutation on the expression of plastid genes, the transcript levels of the plastid-encoded *accD*, *rbcL*, *psbA*, *rrn16*, *rrn23*, and *atpB* genes were also quantified. A reduction only of *rrn16* expression in *anu10-1* mutants was observed, while the expression levels of the other genes were not significantly different from those of L*er* (Fig. 9B), indicating that the defects of *anu10-1* chloroplasts do not result from a general deregulation of organelle transcription.

## Discussion

### ANU10 *encodes a novel plastid protein conserved throughout land plants*

The chloroplast proteome comprises ~1300 different proteins unambiguously identified in proteomic studies (Zybailov *et al.*, 2008; Ferro *et al.*, 2010), ~92% of which are encoded in the nuclear genome. However, the function of a significant fraction (~30%) of these proteins remains unknown (Kleffmann *et al.*, 2004), indicating that additional research is needed to understand fully the processes that take place in the chloroplast. In the present work, the *ANU10* gene has been identified using a map-based cloning strategy. ANU10 is the founding member of a plant-specific family of proteins that contain a chloroplast transit peptide and a transmembrane domain. Using translational fusions to GFP, it has been shown that the ANU10 protein localizes to chloroplasts. A transmembrane domain is also predicted to occur in most ANU10 homologues from other species. In line with this prediction, the ANU10 protein was detected using immunoblotting in the fraction corresponding to thylakoidal membranes, but not in the soluble (stromal) fraction derived from the chloroplasts. Study of the expression pattern and subcellular localization of the ANU10 protein shows that the gene is expressed in a variety of tissues, including cotyledons, leaves, flowers, and roots, and is consistent with microarray data available from the Arabidopsis eFP Browser (http://bar.utoronto.ca/efp/cgi-bin/efpWeb.cgi). In close agreement with this expression pattern, GFP signal corresponding to the ANU10:GFP fusion protein was detected not only in the leaf chloroplasts, but also in the root amyloplasts. The data point to a differential requirement for ANU10 protein in roots and leaves. Although the expression of the full-length coding sequence of *ANU10* restored the wild-type phenotype in *anu10-1* mutant leaves, the root length was not restored to wild-type values, suggesting that roots are sensitive to an excess of ANU10 protein.

### ANU10 *is required for thylakoid biogenesis and grana stacking*

Very little is known as yet about the mechanisms that lead to normal thylakoid biogenesis and grana stacking. The present finding that *anu10-1* mutants exhibit aberrant thylakoid stacking and lack the typical grana seen in normal chloroplasts is expected to help in furthering understanding of these processes. Although a mechanistic link between the molecular function and the observed phenotypes is still lacking, *ANU10* might contribute to the characteristic internal organization of chloroplasts in several different ways.

First, previous authors have proposed that LHCII trimers play a crucial role in thylakoid stacking and grana formation (Day *et al.*, 1984; Allen and Forsberg, 2001; Cui *et al.*, 2011). A lower amount of LHCII trimers in *anu10-1* thylakoids was detected here, in line with the proposed role of trimeric LHCII complexes in thylakoid stacking. Reduced levels of LHCII trimers in the *gdc1-3* mutant have been linked to impaired grana stacking in *Arabidopsis* (Cui *et al.*, 2011), and other authors have shown that normal thylakoid stacking can still occur in plants with reduced levels of LHCB1 and LHCB2 (Andersson *et al.*, 2003), which are compensated by higher levels of LHCB5 (Ruban *et al.*, 2003). Stacks of thylakoidal membranes have been found in other mutants with reduced levels of *LHCB* transcripts, such as *genomes uncoupled1* (*gun1*) (Susek *et al.*, 1993), *immutans* (Wetzel *et al.*, 1994), and *CAB underexpressed4 (cue4)* and *cue9* (Lopez-Juez *et al.*, 1998). Therefore, it is presently unclear if the moderate reduction in *LHCB* transcription and LHCII trimers detected in *anu10* mutants can account for the thylakoid phenotype.

Secondly, the lack of grana stacks in *anu10* mutants might be a consequence of a more general deficiency in thylakoid biogenesis. Thylakoidal membranes, which are less abundant in *anu10-1* chloroplasts, are thought to originate from vesicles that form at the inner envelope of chloroplasts and subsequently incorporate into developing thylakoids (Eggink *et al.*, 2001; Tanz *et al.*, 2012). Three proteins known to participate in vesicle formation and transport during thylakoid biogenesis are VESICLE-INDUCING PROTEIN IN PLASTIDS1 (VIPP1), CHLOROPLAST SECRETION-ASSOCIATED RAS1 (CPSAR1), and SNOWY COTYLEDON2/SHI-YO-U1 (SCO2/CYO1). The VIPP1 protein localizes to the thylakoidal and chloroplast inner membranes, and the *high-chlorophyll fluorescence155* (*hcf155*) mutant shows that VIPP1 is required for the formation of the vesicles (Kroll *et al.*, 2001). CPSAR1 dually localizes to the chloroplast stroma and the inner envelope, and has been proposed to participate in the initiation of vesicles from the inner envelope (Garcia *et al.*, 2010). SCO2/CYO1 is required for the trafficking of vesicles from the inner envelope to developing thylakoids (Tanz *et al.*, 2012). Interestingly, the *anu10-1* phenotype resembles the phenotype of loss-of-function *hcf155*, *cpsar1*, and *sco2-1* mutants as regards the low abundance of thylakoids (Kroll *et al.*, 2001; Garcia *et al.*, 2010; Tanz *et al.*, 2012). This raises the possibility that ANU10 participates in thylakoid biogenesis by modulating vesicle integration into developing thylakoids. Under this scenario, the reduced levels of LHCII trimeric forms detected in *anu10-1* thylakoids would simply be an indirect consequence of inefficient thylakoid biogenesis, as LHCB proteins have been proposed to be targeted to thylakoids, at least in part, through their incorporation into developing vesicles in the inner membranes of chloroplasts (Eggink *et al.*, 2001; Tanz *et al.*, 2012).

Because some proteins involved in plastid gene expression, such as pTAC14 (Gao *et al.*, 2011) or the nucleus-encoded RPOTmp polymerase (Azevedo *et al.*, 2008), are associated with the thylakoidal membranes, and *ptac14* mutants have been reported to lack grana and thylakoidal membranes (Gao *et al.*, 2011), it was also considered here that the defects in thylakoid abundance and stacking might result from a more general problem in plastid gene expression in *anu10-1*. However, the results of the present study indicate that the expression of plastid genes is not generally affected.

Leaf senescence has also been associated with thylakoid defects in higher plants (Evans *et al.*, 2010; Krupinska *et al.*, 2012). In the *anu10-1* mutant, however, the senescence marker *ORE1* was expressed at normal levels, suggesting that senescence is not the primary cause of the observed plastid phenotypes.

### Loss of ANU10 function compromises leaf development

Altered leaf anatomy is a trait common to many mutants carrying lesions in nuclear genes that encode plastid proteins. Some examples are the *immutans* (Wetzel *et al.*, 1994; Carol *et al.*, 1999; Aluru *et al.*, 2001), *cue1* (Li *et al.*, 1995; Streatfield *et al.*, 1999), *pale cress1* (*pac1*) (Reiter *et al.*, 1994), and *rugosa2* (*rug2*) (Quesada *et al.*, 2011) mutants of *Arabidopsis*, the *differentiation and greening* (*dag*) mutant of *Antirrhinum majus* (Chatterjee *et al.*, 1996), and the *defective chloroplasts and leaves* (*dcl*) mutant of tomato (Keddie *et al.*, 1996). These mutants display variegated or pale-green phenotypes and have altered plastid and mesophyll development. In some variegated mutants, such as *rug2*, the pale sectors exhibit severe developmental defects in plastids and mesophyll cells, while the cells and chloroplasts within green sectors are usually less affected (Quesada *et al.*, 2011).

Because some mutations damaging plastid-localized proteins affect both plastid development and mesophyll cell differentiation, plastid development and leaf morphogenesis have been hypothesized to be tightly coordinated processes via retrograde plastid-to-nucleus signalling (Rodermel, 2001). The signal is assumed to originate from chloroplasts with arrested differentiation or altered photosynthetic metabolism, and modulates the expression of nuclear genes encoding plastidial and other proteins (Susek *et al.*, 1993; Koussevitzky *et al.*, 2007). Pyke *et al.* (2000) proposed that a cell-autonomous signal originating in the plastids is required for normal development of palisade mesophyll cells. Like the above-mentioned mutants, the *anu10-1* mutant exhibits altered mesophyll cell development, with larger and less densely packed palisade cells. The present morphometric analysis shows that *anu10-1* leaves have fewer, larger mesophyll cells per area unit, suggesting either a premature transition from proliferation to cell expansion, or a defective cell proliferation. The increased extent of expansion observed in *anu10-1* is in contrast to previous studies (Andriankaja *et al.*, 2012), which have shown a delay in mesophyll cell differentiation when chloroplasts are prevented from differentiating. Different chloroplast-to-nucleus signals acting in differentiating and mature chloroplasts might explain the different effect on mesophyll cell expansion.

*LHCB* genes, among many other nuclear genes related to plastid photosynthetic machinery and other diverse functions, are known to be differentially expressed in response to chloroplast retrograde signalling (Susek *et al.*, 1993; Koussevitzky *et al.*, 2007). Furthermore, recent experimental evidence suggests that retrograde signalling also modulates the expression and activation of cyclin-dependent kinases, which are key regulators of the cell cycle (Kobayashi *et al.*, 2009; Andriankaja *et al.*, 2012). The defects of *anu10-1* chloroplasts might trigger a retrograde signal leading to changes in nuclear gene expression, as exemplified by the reduced expression of *HEMA1* and the *LHCB1*, *LHCB2*, and *LHCB3* genes.

### Concluding remarks

A nuclear gene, *ANU10*, has been identified, whose loss-of-function mutations lead to pleiotropic defects in leaf development and plastid internal organization, including a dramatic reduction in thylakoidal membranes and grana stacking. The results indicate that these phenotypes are not merely a consequence of a premature senescence response or of a general defect in the transcription of plastid-encoded genes. The expression of several nuclear genes, including *HEMA1* and several *LHCB* genes, was found to be altered in the *anu10-1* mutant, suggesting that the plastid defects trigger a retrograde (chloroplast-to-nucleus) signal that might account for the mesophyll phenotype.

## Supplementary data

Supplementary data are available at *JXB* online.

Figure S1. Growth of the *anu10-1* mutant.

Figure S2. ROS in *anu10-1* leaves.

Figure S3. Rosette phenotype of homozygotes and heterozygotes for the *anu10* alleles.

Figure S4. Phylogenetic analysis of ANU10 and its putative orthologues.

Figure S5. Subcellular localization of the ANU10:GFP fusion protein in the *anu10-1 ANU10*_*pro*_*:ANU10:GFP* transgenic line.

Figure S6. Phenotypic rescue of *anu10-1* by the ANU10:GFP fusion protein.

Figure S7. Growth and starch content of *anu10* roots.

Table S1. Primer sets used in this work.

Table S2. Transmembrane domains and chloroplast transit peptides in the ANU10 protein and its putative orthologues.

Supplementary Data
