# Supplementary material for: Arabidopsis ANGULATA10 is required for thylakoid biogenesis and mesophyll development
Source: J Exp Bot. 2014 Mar 24;65(9):2391–404. doi: 10.1093/jxb/eru131 (PMC4036511; doi:10.1093/jxb/eru131)
Supplement: Supplementary Data [file supp_65_9_2391__index.html]

 Arabidopsis ANGULATA10 is required for thylakoid biogenesis and mesophyll development — Arabidopsis ANGULATA10 is required for thylakoid biogenesis and mesophyll development — Supplementary Data 

# *Arabidopsis* ANGULATA10 is required for thylakoid biogenesis and mesophyll development

## Supplementary Data

Data files

**Files in this Data Supplement:**

- Supplementary Data - Supplementary Data
