# Supplementary material for: Arabidopsis ANGULATA10 is required for thylakoid biogenesis and mesophyll development
Source: J Exp Bot. 2014 Mar 24;65(9):2391–404. doi: 10.1093/jxb/eru131 (PMC4036511; doi:10.1093/jxb/eru131)
Supplement: Supplementary Data [file supp_eru131_Casanova_S_ez_et_al_Supplemental_data.pdf]

Journal of Experimental Botany

Arabidopsis ANGULATA10 is required for thylakoid biogenesis and mesophyll development

Rubén Casanova-Sáez, Eduardo Mateo-Bonmatí, Saijaliisa Kangasjärvi, Héctor Candela and José Luis Micol

Supplementary Data

Author contributions

H.C. and J.L.M. conceived and designed the research. R.C.S., E.M.B., S.K. and H.C. performed the research. R.C.S, H.C. and J.L.M. wrote the article.

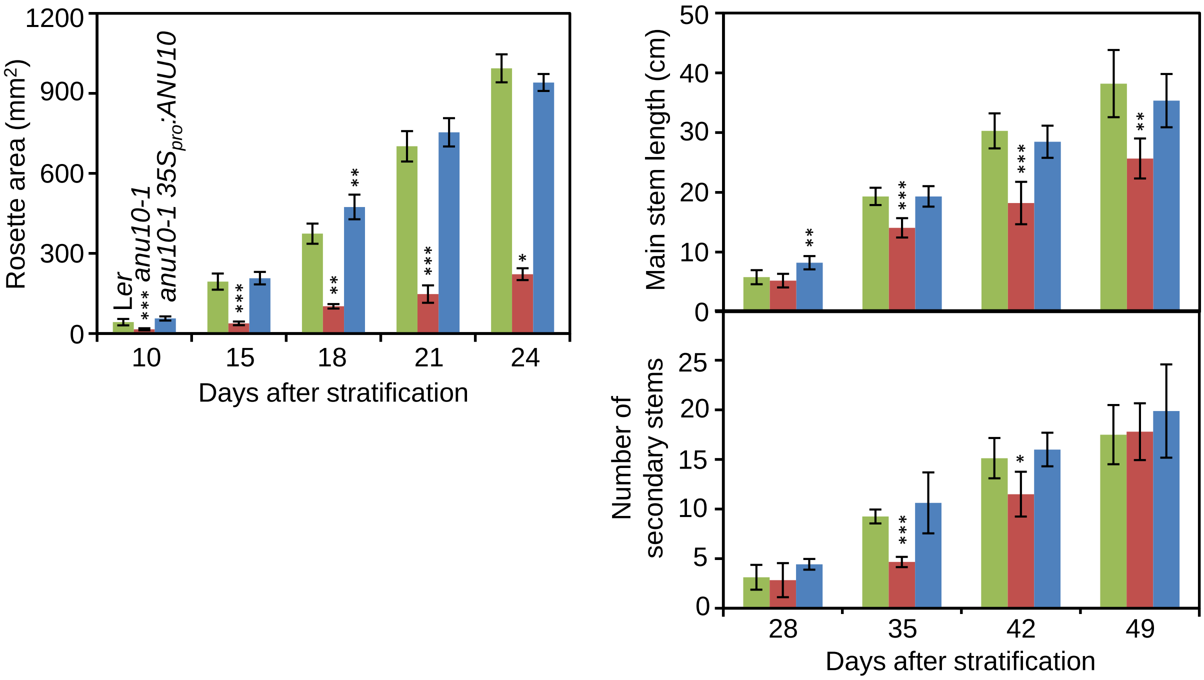

Supplementary Figure S1. Growth of the *anu10-1* mutant. Error bars indicate standard deviations. Asterisks indicate values significantly different from L*er* in a Mann-Whitney U test (*p < 0.05, **p < 0.01, ***p < 0.001; n = 8).

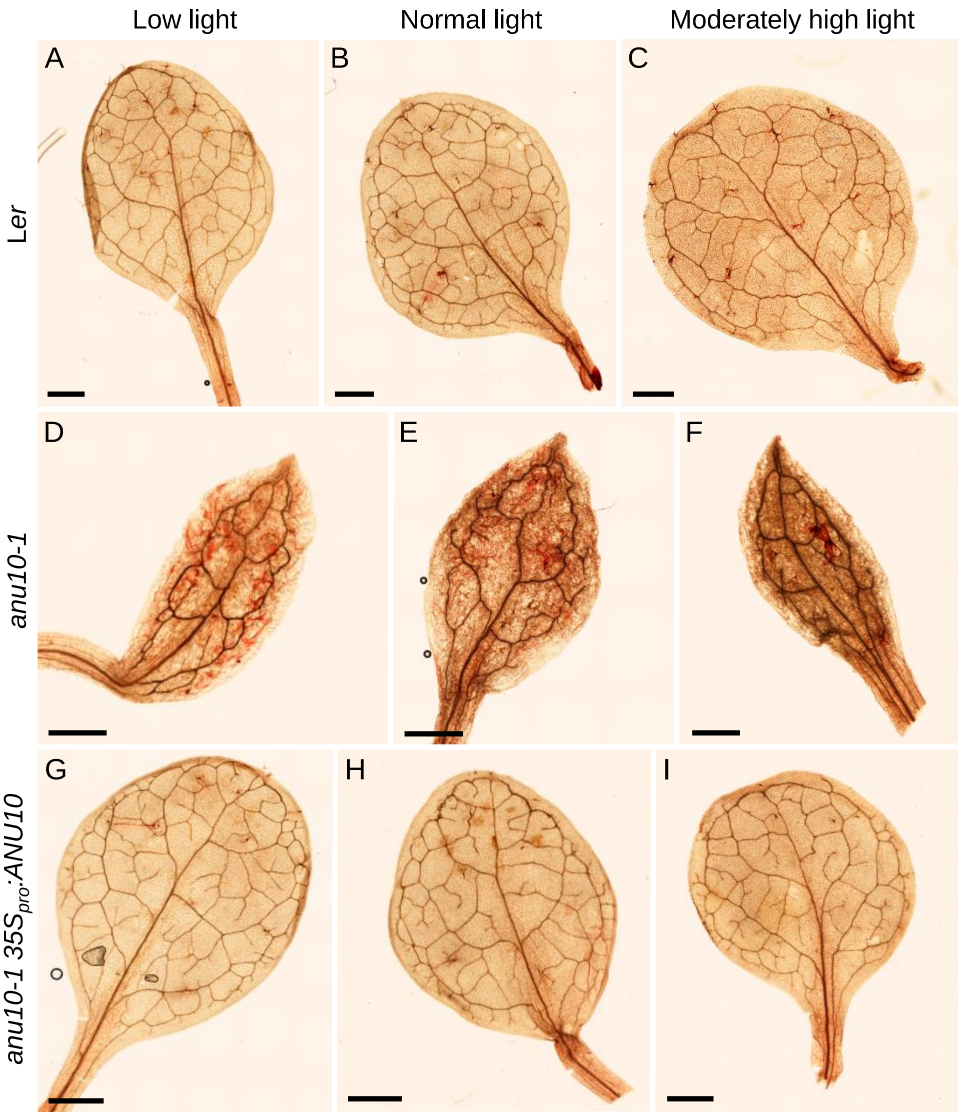

Supplementary Figure S2. ROS in *anu10-1* leaves. H_2O_2 accumulation visualized by DAB staining in first-node leaves from (A-C) L*er*, (D-F) *anu10-1* and (G-I) *anu10-1 35S*$_{pro}$*:ANU10* plants collected 16 das. Plants were grown at low (~55 µmol m^{-2} s^{-1}), normal (~90 µmol m^{-2} s^{-1}) or moderately high (~180 µmol m^{-2} s^{-1}) light intensity. Scale bars indicate 1 mm.

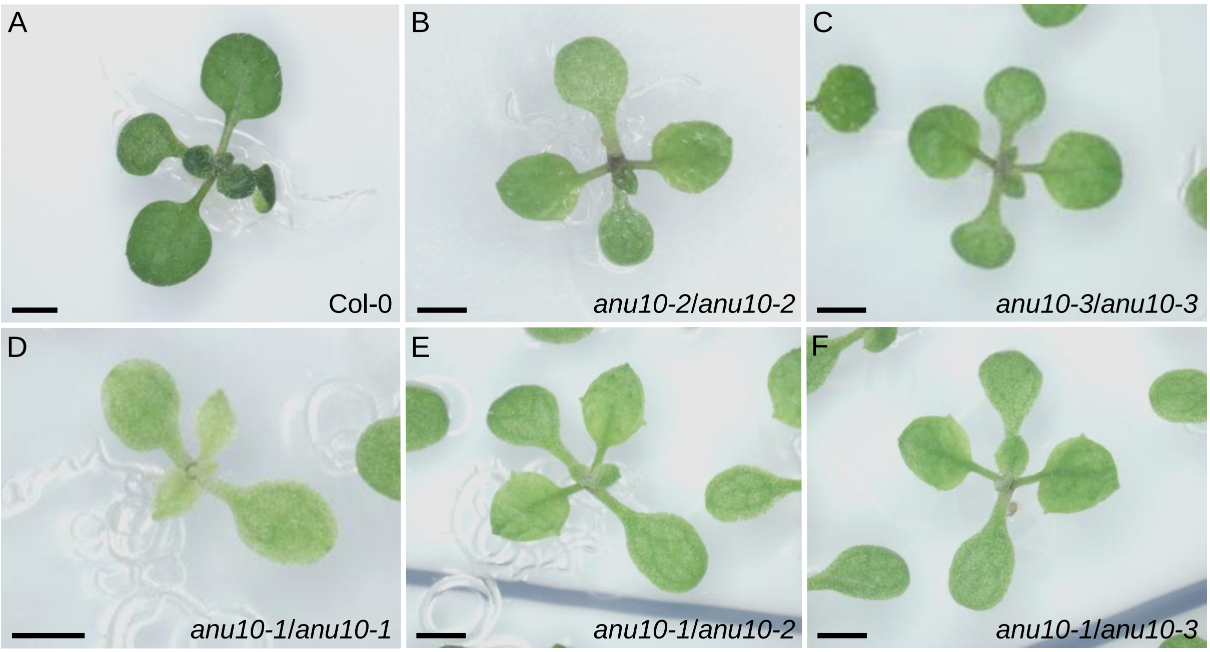

Supplementary Figure S3. Rosette phenotype of homozygotes and heterozygotes for the *anu10* alleles. Pictures were taken 10 das. Scale bars indicate 2 mm.

A

```
Ppa XP_001764411.1     1 -------------MALSSGSQIVLPYSQSSSGIPWGCFLPRHHARHSQAIRKLQLL[illegible]DLI
Sbi XP_002451363.1     1 --------------------------MPSPSLLLRALPLCPSPSNSHS---PRRFAPSPL
Osa BAD07831.1         1 --------------------------MPSPSLLLHHHHHRLSPRFAPS---R---SPSPR
Ata EMT06249.1         1 ------------------------------------------------------------
Tur EMS59110.1         1 --------------------------------MEWSGSLSRSP-----------------
Sly XP_004234099.1     1 --------------------------MAFTLTLINSPANSISPETNSAIRKCPLFIRNLH
Cru EOA37571.1         1 -------------------------MASTASSSLLFPSRNFKS------------VPLIP
Ath NP_174175.2       1 -------------------------MASISTASSSSSLLFPSRNFKSI----------PL
Aly XP_002893534.1     1 -------------------------MASVSTASCSSSSSLLFPSRNFA------SIPLIP
Csa XP_004134942.1     1 --------------------MAISPMALSSYFIQYSPIQTIPARRLFA--IPSIYTPKIV
Gma XP_003535421.1     1 ------------------------MTMAIAASSLSSPVLSY---TKFS-------NPTPR
Gma XP_003541453.1     1 ----------------------------MAASSLFSPVLPYTKFSSST----------LR
Mtr XP_003593674.1     1 -----------------------MASVTSTTTTLFTPFLSHRHTTKPF-----LFSPPLH
Car XP_004485849.1     1 MKCPAQQEGTQHFSLSLSPMATAMATVATSTTSLFTPFVPLRSNSKFS-----LFFPTLH
Rco XP_002522507.1     1 --------------------------MASHSLSLFSSVFLSPPQSLSR-----TPIPKLF
Tca EOY28124.1         1 -------------------------MAFAHSVTLLSPAFPTQFPSCFP--RKLPFFPTLP
Vvi CBI30631.3         1 -------------------------MAFIHPPALRIPLLTFQPRSSST--RILTFFPKLL
Vvi XP_002273236.2     1 -------------------------MAFIHPPALRIPLLTFQPRSSST--RILTFFPKLL
Fve XP_004295562.1     1 -------------------------MTSAHSLLLHPPLVSFTRPSRCS-----------L
Ppe EMJ12593.1         1 ----------------------MCPGERSGSSGNQNDVVPF-------------------
Consensus              1                               .  . .. .     .           . .

Ppa XP_001764411.1    48 LHASSCSVRRGGKQRALSKSIRGKSAENSAQYEDTRIDTEAGQSWNYAATRTYRKAMASL
Sbi XP_002451363.1    32 TTSSPADRLRPLRLTPAASRHTPRTSVRAVV----------DGGDLIPIARCYEGRLARL
Osa BAD07831.1        29 TLSLHPAPRLRLRLRLRASARGGGGSTRDHD----------DGARSIPIARCYEAALARL
Ata EMT06249.1         1 ------------------------------------------------------------
Tur EMS59110.1        12 -----------------------------------------GKGASPHAVRRLHADRAAA
Sly XP_004234099.1    35 FTLYRNRRRS-ISCSAQQKPFKPMTEENILEA----VADFDGDEKSVPCLRTYENDIARL
Cru EOA37571.1        24 LRRRSFVSFIRFSKN-------PSAEEDILRF----VAE--SDGMALPCVRTYENNSARL
Ath NP_174175.2      26 LPQQRSSVSF-VRCFSKN----SSTEEDILRF----VAE--SDGKALPCVRTYENNSARL
Aly XP_002893534.1    30 LRRSSVSFIRGYSKN-------SSTEEDILRF----VAE--SDGKALPCVRTYENNSARL
Csa XP_004134942.1    39 LKNSRHCFST-FSCSAGRPI--PTTEEEVLQA----VLE--SDEKILPCVRTYENDLSRL
Gma XP_003535421.1    27 FTNSKNSIFA-LSCSSPKSI--PVTEKEVLQA----IAD--SDGKNLPCVRTYDTDLSQL
Gma XP_003541453.1    23 FTNSKNSIFA-LSCSSPKSI--PVTEKEVLQA----IAD--SDGKNLPCVRTYDNDLSQL
Mtr XP_003593674.1    33 LPNTTPSIFI-LSCSPPKTI--PVTEQQFLQA----ISD-SSDQKQLPCVRTFENDLSQL
Car XP_004485849.1    56 FPNSKYSIFV-LSCSPPKTI--PVTEQQVLKA----IAD-SSDQKRLPCVRTFENDLSQL
Rco XP_002522507.1    30 LTNARNCIPV-LSCT-------PPQQEAILQL----VAN--ADENTLPCIRAFENDLARV
Tca EOY28124.1        34 FRNPTVPLLI-LSRSSPKPA--PSTEPEALKF----ITD--SDGITLPCVRTYENDLARL
Vvi CBI30631.3        34 FKNPRNSLYI-VSCSTPKAI--PATEQEVLDA----IAE--SDEKSLPAVRSFENDLARL
Vvi XP_002273236.2   34 FKNPRNSLYI-VSCSTPKAI--PATEQEVLDA----IAE--SDEKSLPAVRSFENDLARL
Fve XP_004295562.1   25 FSTPRNSRFTLACCSSRKTA--PVTEQEVLQA----ISE--SAEKSLPCVRTYENELARL
Ppe EMJ12593.1        20 -------------------------QQEVLQA----VAD--SDERVLPGVRTYENDSACL
Consensus            61   . . ..   ....       . .. ... .    ...  .. . ..............

Ppa XP_001764411.1   108 RVTGNALPLQVN-AALAADGGRTAVQELKQGKAMLVIEAYNPGQ-LSEGSTVSTKLQAPT
Sbi XP_002451363.1    82 ELAGAARREQAVAAAAAADGGARAEAYLAAGSDAMVVEAFLPGSHGGGTTASSTRVILQA
Osa BAD07831.1        79 DLSGTARREQAVAAAAAADGGAAAEAHLAAVADAMVMEAFLPGPDGARRSAMSTRLILQA
Ata EMT06249.1         1 ----------------------------------MVMEAFLPGA----GGAASTRVILQA
Tur EMS59110.1        31 NFGHTEAPRMSV-LQMRKRWGREDKGSARVALRAMEI--------------------LQA
Sly XP_004234099.1    90 TLVGAVDFQQAL-TAAAADGGEAAGEHIAAGMDAMVVETLFPGP-SDEHSTISTRLFLPA
Cru EOA37571.1        71 SLVGTVAFDQAL-TAAAADGGEAADDHLRENVPVMVVETVFPGG-SDPKATVSTRLFLPT
Ath NP_174175.2      75 SLVGTVAFDQAL-TAAAADGGEAADDHLRENVPVMVVETVFPGG-SDPKATVSTRLFLPT
Aly XP_002893534.1    77 SLVGTVAFDQAL-TAAAADGGEAADDHLRENVPVMVVETVFPGG-SDPKATVSTRLFLPT
Csa XP_004134942.1    90 SLVGGVDFRQSV-TAAAADGGEAATEHLDSGMSAMVVETVFPGT-SDEHSTVSTRLFLPA
Gma XP_003535421.1    78 TLVGTVDFHQAL-TAAAADGGEVATDHIDAGLDAMVVETVFPAP-SCDHATVSTRLFLPA
Gma XP_003541453.1    74 TLVGTVDFNQAL-TAAAADGGEVASDHIDAGLDAMVVETVFPAP-SSDHATVSTRLFLPA
Mtr XP_003593674.1    85 TLVGAVDFRQAV-TAAAADGGEVADEHVQAGMDAMVVETVFPAS-SSDHGTVSTRLFLPA
Car XP_004485849.1   108 TLVGAVDFRQAV-TAAAADGGEVASEHIDASMDAMVVETVFPAS-SSDHGTVSTRLFLPA
Rco XP_002522507.1    76 SLVGSVGFDQAV-VAAAADGGRAAADHIDSGAPAMVVETLFPGP-GDHHATISTRLFLPA
Tca EOY28124.1        85 TLVGAVGFEQAL-TAAAADGGRAATEHVDSGIPAMVIETVFPSS-IAKNATVSTRLFLPA
Vvi CBI30631.3        85 TMVGAIDVEQAL-TAAAADGGNTADEHIASGMAAMVVETVFPGA-SDEHSTVSTRLFLPA
Vvi XP_002273236.2   85 TMVGAIDVEQAL-TAAAADGGNTADEHIASGMAAMVVETVFPGA-SDEHSTVSTRLFLPA
Fve XP_004295562.1   77 GVVGAVDFQQAL-TAAAADGGEAADEHIREGVPAMVVETLLPGN-SDPYSTISTRLFLPA
Ppe EMJ12593.1        49 ALVGAVDFEQAL-TAAAADGGQAADEHISSGMPAMVVETLFPGH-SDPHSTVSTRLFLPA
Consensus           121 ............ ...............  .. ..........  .. ............
```

```
Ppa XP_001764411.1   166 LAVIERA-KIMAEKDAQFLRGGR-----AFAVAFQTVRLLKLSKFELCFYQPGTMKATDG
Sbi XP_002451363.1   142 REVKDKASKIEKQFGYDFFFANESDSESMLAMAFKQVVIQRLSNERLEVFSPGSVRHFQD
Osa BAD07831.1       139 NEVTEKASKLKKDLGTEFFSENEPDAESVLAMAFKQVVMDQLTNERLEVFSPGSEIDLQD
Ata EMT06249.1        23 KEVKEKAAKIKKDFGDDIFSENEPDSESILAMALKQVVMHKLSNYRLEIFSPGSGRDFQD
Tur EMS59110.1        70 KEVKEKAAEIKKDFGDDIFSENEPDSESILAMALKQVVMQKLSNYRLEIFSPGSGRNIQD
Sly XP_004234099.1   148 RKVMEKAHKLRSTITKEMLSGTT--STNILAMTFRQVTLQHLWNFDLVLFIPGTERNMDD
Cru EOA37571.1       129 KKVKERAKKLRRSLSEDLSSGDL--SKNILAMTFRQVVLRQLWNFQLVLFGPGAEREMGD
Ath NP_174175.2      133 KKVKERAKRLRRSLSEDLSSGDL--SKNILAMTFRQVVLRQLWNFQLVLFAPGAEREMGD
Aly XP_002893534.1   135 KKVKERAKKLRRSLSEDLSSGDL--SKNILAMTFRQVVLRQLWNFQLVLFAPGAEREMGE
Csa XP_004134942.1   148 RKVREKATKLRKSLAQDFNSSTE--SKNILAMTFRQVVLQQLWDFELVVFTPGSDRNMED
Gma XP_003535421.1   136 RKVKEKAAKLRNSFPKDLFSGSG--SKNVLAMTFRQVVLQQIWSFDLTVFQPGEERKMVD
Gma XP_003541453.1   132 RKVKEKATKLRKSFPKDAFSGSA--SKNVLAMTFRQVVLQQIWSFDLTVFQPGEERKMDD
Mtr XP_003593674.1   143 RKVKEKAAKLRKSFSEDIFSNTT--SRNVLTMTFRQVVLEQVWNFDLTVFQPGEERKMDD
Car XP_004485849.1   166 RKVKEKAAKLRKSLSQDMFSNTT--SRNVLAMTFRQVVLEQIWNFDLIVFQPGEERKMED
Rco XP_002522507.1   134 KKVKEKAAKLSRSFKEDIFSGTA--SENILAMTFRQVVLQQLWNFELAVFRPGTERNMED
Tca EOY28124.1       143 RKVKEKAQKLRKSLSEDILTETS--SKNILAMTFRQVVIQKFWNFELVLFRPGTERDMED
Vvi CBI30631.3       143 RKVKEKANRLRRSFTEDFLSSTT--SKDILAMTFRQVVLQQLWNFELVLFIPGTERNMEE
Vvi XP_002273236.2   143 RKVKEKANRLRRSFTEDFLSSTT--SKDILAMTFRQVVLQQLWNFELVLFIPGTERNMEE
Fve XP_004295562.1   135 RKVKEKAGKLRSSITQDMLSTTT--SRNILAMTFRQVVLELLWNFQLVVFRPGTQRNMKD
Ppe EMJ12593.1       107 RKVKEKAGKLRRSITEDMLSTAT--SRNILSMTFRQVVLQLLWNFELVIFRPGTERNMED
Consensus            181 ..*...* .......... . .   ..........*.........*...  **... ...

Ppa XP_001764411.1   220 YG-----GEIAAFLEVSDASCLSEIAKGVCSYALACVKDNAEQRAKVEGFGSIP------
Sbi XP_002451363.1   202 FGKPQK-GSLDCSISSSNGKLLSSLAEAIFYCVI------EDARKNHLGGVGS--------
Osa BAD07831.1       199 LSKPRK-VPADFSIRSSDEKLLAALAEAIFSCVI------EDARNNYLGGTGG--------
Ata EMT06249.1        83 WSKPRK-VPVDFSISSSDGKLLSALAEAIFSCVI------EDTEKSYFGGTGG--------
Tur EMS59110.1       130 WSKQRK-VPVDFSISSSDEKLLSALAEAIFSCVI------EDTEKSYLGGTGG--------
Sly XP_004234099.1   206 LETPRE-VPPSFAIKSSDERVISVIAEVICLSAL------ESTKKHEFNGTPGGAPKKFFR
Cru EOA37571.1       187 FENPRE-VSTSFTLSSTDERVISVIAEVLCISAL------QSTEKHFLDDYLGKAKFPFFK
Ath NP_174175.2      191 FENPREQVSTSFTLSSSDERVISVIAEVICISAL------QSTEKHFLDDYLGKAKFPFMK
Aly XP_002893534.1   193 FENPREQVSTSFTLSSSDERVISVIAEVICISAL------QSTEKHFLDDYLGKLKFPFLK
Csa XP_004134942.1   206 LENPRE-VPISFTLSSSEERAISVLAETVCMCAL------QNTEGKFVDGTSRGTSTRLFG
Gma XP_003535421.1   194 LETPRE-VPASFALSSADEYLISVLAEAICISAL------QSTQIQFLEKVKGGNRGGFFR
Gma XP_003541453.1   190 LETPRE-VHASFALSSSDEYLISVLAEAICISAL------QSTQIQFLDKAKGGNRGGFFR
Mtr XP_003593674.1   201 LENPRE-VPASFTLSSSDEYLISMLAEVVCISSL------QSTQRQFLDKSQDGSRSGFFQ
Car XP_004485849.1   224 LENPRE-VPASFTLSSSDEYLMSVLAEVVCISAL------QNTQRQFLDKSQGGSRSGFFR
Rco XP_002522507.1   192 LENPRE-VPASFFLSSSDEHIISVLAEAIYIAAL------QNTESNFLYDFMGETSGGVFR
Tca EOY28124.1       201 LENPRK-VIGSFTLSSSEERVISNLGEVVCISAL------QSTERHFLDNFLGKTSSNFFQ
Vvi CBI30631.3       201 LQDSRKQVPASFFLSSSDEGVISVLAEVICISAL------QTTERHFINNLLGQTSNNFFK
Vvi XP_002273236.2   201 LQDSRK-VPASFFLSSSDEGVISVLAEVICISAL------QTTERHFINNLLGQTSNNFFK
Fve XP_004295562.1   193 LENARE-VPACVTLSSSDERILSVLAEVVCISAL------QNTEKQFLEDYTGKTPSDLLH
Ppe EMJ12593.1       165 LENPRE-VPPSFTLSSSDERIMSVLAEVVCISAL------ENTERQFLEDFMGKTKSNLVR
Consensus            241 ...... ...........................      ..... ...   .    ...

Ppa XP_001764411.1   269 FLKRSEGIKTTVKNSTVQISSFTEAEVSAHVERIVQDKKLVGTSVGLRRHRS---WWNRP
Sbi XP_002451363.1   248 LFQKRQLDYS--MDSSVCIHRISEAEVVKNAKRCLETISLIKSSHKVHETKNG--WWPPP
Osa BAD07831.1       245 LFHKWKSNCS--LDSSVCIDIISESEVVNSARRRLDSFDLVQSSHVAGKAKNG--WWPAP
Ata EMT06249.1       129 FFQSQKLNCS--SDSTVCVHRISEAEVANNARRCLESFNLTKSSHEVGRLKNV--WWPAP
Tur EMS59110.1       176 FFQTQKLNCS--SDSTVCIHRISEAEVANNARRCLESFNLTKSSHEVGRSKNA--WWPAP
Sly XP_004234099.1   260 WFDTHKSIVS--KDSSVTLYNLMEYEIVANANVLLQKFSSERANYRPREGRWMGNWLTST
Cru EOA37571.1       241 WFSKHKRIAS--RDSSVVLHKVFDDE--QNTNHLLEYYQSREEKFKLADTRQRSRWWNMS
Ath NP_174175.2      246 WLSKRRRIAS--RDCSVVLHKLFDDE--QNTKLLLEYYQSRKENFKLADIKQRSRWWDLS
Aly XP_002893534.1   248 WLNKHKRIAS--RDCSVVLHKLFDDE--PNTNLLLEYYQSRKENFKLEDTRQRSRWWNLS
Csa XP_004134942.1   260 WFRKSTIVAS--KDSSVVIHKLFDNEI-ADPKSLLQKFNSNKESWKHRNSKSMNYWWMPT
Gma XP_003535421.1   248 WFRKPESVQS--KDSAVILSKLFEDEIVENARSLLDNYNLMKDGFKPVKIKSGHHWWKSS
Gma XP_003541453.1   244 WFQKPESVQS--KDSAIILSKLFEDEIVENARSLLDNYNLMKDGFKPVKIKSGHHWWKPS
Mtr XP_003593674.1   255 WFQKHERIQS--KDSAVILHKLFEDEIVENARSLLDKYHLMKDGFKPVKIKSERFWWKPS
Car XP_004485849.1   278 WFQKPERIQS--KDSAVILHKLFEDEIVENAKSLLDNYHLMKDGLKPVKIKSGPFWWKPS
Rco XP_002522507.1   246 WFQKPKSIAS--QDSSVVIYKLFEDEIAGNAKKLLEDFNLSKD--KGIKLRQKYKWWTPV
Tca EOY28124.1       255 WFRKSKAIES--KDLSVVIYKMFEDEIVENAKSLLESFNSSEESFKTMDLRRKDYWWTPS
Vvi CBI30631.3       256 WFHKPKSVAS--KDSSVIMYELFEDEIVENAKNLLENFNSMKANYKCIETKTKYHWWTSS
Vvi XP_002273236.2   255 WFHKPKSVAS--KDSSVIMYELFEDEIVENAKNLLENFNSMKANYKCIETKTKYHWWTSS
Fve XP_004295562.1   247 SFRKPRRIVS--KDSSVVIYKLFEDEIVENAKSLLEHFNSAKNSFKSVKTKSK--WWTPS
Ppe EMJ12593.1       219 WFRKPKRTVS--KDSSVVIYKLFEDEIVENAKSLLKNFNLTKGSFKPVKTKSKYYRWTAT
Consensus            301 .. . . .   ....... .....*.. ... ... ... .. .. .....   ..  .
```

```
Ppa XP_001764411.1  326 LATEAVVKSGLD-AQLVDEFLPAHKLQLN---LPSNKFEAVGLQKTSDTVCSINLTHAQM
Sbi XP_002451363.1  304 HYETLVKIGGPELVLWANEYIPTYKLQINANALENSNHE--GLFELESNRWEVLLSHSQL
Osa BAD07831.1       301 KSERLVKIGGPDFMLWASEFVYTYKLQIDAKAFKNTKLG--GHHVLANNKGEVLLSHAQM
Ata EMT06249.1       185 KYGRLAEIGSPDFILWAHEFVPSYKLQINANAFENTKLE--GCHELVNNRWEVPISHFQL
Tur EMS59110.1       232 KYGRLAEIGGPDFILWAHEFVPSYKLQINANAFENTKLE--GCHELANNRWEVPISHFQL
Sly XP_004234099.1  318 TYFKLEQIGGPEFISWLSERVPAYKLQIDAEKLDNVKFE--GWKETATNTWEVFLTHSQM
Cru EOA37571.1       297 ANSKLEKFGGPGFSTWASEYLPAYRLEIDTTILADLKLE--GWRKSSENKWEVLLTHSQM
Ath NP_174175.2      302 ANSKLEKIGGPGFSSWASEYLPAYRLEMDSTILADLKLE--GWRKSSENKWEVLLTHSQM
Aly XP_002893534.1  304 ANSKLEKIGGPGFSSWASEYLPAYRLEIDTIILADLKLE--GWRKSSENKWEVLLTHSQM
Csa XP_004134942.1  317 ELTRLEKIGGSEFCAWVSEYVPAYRLQIDAHQFNGLKFG--GWREFVENRWEVLLTHSQM
Gma XP_003535421.1  306 CYEKLEKIGGSDFSAWASEYVPAYRLEIDTKILGDSKIG--GWKKSAENRWEVLLTHSQM
Gma XP_003541453.1  302 CYEKLEKIGGSDFSAWASEYVPAYRLEIDTKIVGDSKIE--GWKKSAENRWEVLLTHSQM
Mtr XP_003593674.1  313 SYEKLEKIGGSDFSAWTSEYVPAYRLEIDPKIMGDSKFQ--GWKKSSENRWEVLLTHSQM
Car XP_004485849.1  336 CYEKLEKIGGSDFSAWTSEYVPVYRLEVDTKIMGDAKFQ--GWKKSSDNRWEVLLTHSQM
Rco XP_002522507.1  302 ALSKLETIGGSDFSAWVSEYVPAYRLQIDADIVKDAKME--GWRRFGDNRWEVLLTHSQM
Tca EOY28124.1       313 MHSKLDKIGGLEFSAWTSEFIPAYRLEINANTLKDLKFE--GWIKSDENRWEVLLTHSQM
Vvi CBI30631.3       314 AISKLEKIGGPEFSTWTSEYIPAYRLQIDPDKLKSVKFE--GWKRSAENRWEVLLTHSQM
Vvi XP_002273236.2  313 AISKLEKIGGPEFSTWTSEYIPAYRLQIDPDKLKSVKFE--GWKRSAENRWEVLLTHSQM
Fve XP_004295562.1  303 ALSKLEKIGGPEFSCWTSEYVPAYKLQIDANRHKDVKFE--GWRKCGETSWEVLLTHSQM
Ppe EMJ12593.1       277 ALSKLEKIGGPEFSSWTSEHVPAYRLQIDANQHKDVKFE--GWRKSAENWCEVFLTHSQM
Consensus           361    ............ . .*......*....  . .....  *..............*.*.

Ppa XP_001764411.1  382 MDLADVLDLYSGDPYTGSGESVSMSL------DIPRR-----SWAKVVQ-TVAVGALATA
Sbi XP_002451363.1  362 VELGNILDMYFEDQFTLPGKTF-----HPHWNSDPSKIKKNNGYLNNLFTFLAGSCIFLF
Osa BAD07831.1       359 VELANILDMYFEDQFTLPGKTF-----YSNWNSEPSKIKKNNGYLNNLFALLAGSSIVFL
Ata EMT06249.1       243 VELGNVVDMYFEDQFTIPGKTF-----RSHWNAEPSKIRRNNGYLNNLFSFLAGCSVIFI
Tur EMS59110.1       290 VELGNVVDMYFEDQFTIPGKTF-----RSHWNAEPSKIRRNNGYLNNLFSFLAGCSVIFI
Sly XP_004234099.1  376 VGLSDVLDMYYEDVYTLPYKQLSLGV-VAKSFNSPSS-KRSISMSKAISMVLASGIFLVA
Cru EOA37571.1       355 VGLAEALDIYFEDIYSLPKKQLPCDV-SGNYANLPNE-KRGLSLLKIISVTMASGILLLA
Ath NP_174175.2      360 VGLAEALDIYFEDTYSLPRKQLPCDV-PGNYANLPNE-KKGLSLLKFISVTMASGIFLLA
Aly XP_002893534.1  362 VGLAEALDIYFEDIYSLPRKQLPCDV-PGNYANLPSE-KRGLSLLKFISVTMASGILLLA
Csa XP_004134942.1  375 VGLANILDMFYEDVYSLPDKQLQCGA-NVHSANLLSK-KRNYSSWGLLSKTLAGGVFLVA
Gma XP_003535421.1  364 VQLAETLDIYYVDPYSLPNKQLSCGV-AAKFANVSNK--KGNSFPKFLSLALASGIFLVA
Gma XP_003541453.1  360 VQLAETLDIYYVDPYSLPYKQLSCGV-AAKFANVYNK--KGNSFPKLLSFALASGIFLVA
Mtr XP_003593674.1  371 VGLAETLDMYYVDPYSLPDKELSYGV-AAKYANVSNR--KGSSLSKILSVSLASGMFLVA
Car XP_004485849.1  394 VSLAETLDMYYVDPYSLPDKDLSCGV-VAEYVNVSSR-KVRLYLSKFLSVTLATGMFLVA
Rco XP_002522507.1  360 AGLAEILDMYFEDVYSLPHKELSCRA-TANFTNLANT-KKSSSWLKFLSVSLISGIFLIA
Tca EOY28124.1       371 VGLADILDLYYEDVYSLPNKQLRCGV-VANYNSLSKA-KRSSSFSRMVSATVASGILLIT
Vvi CBI30631.3       372 VALANILDMYYEDLYTLPDKQLLCGAGFANFTNLSKN-KRISSLLKILTISIASGIFLVA
Vvi XP_002273236.2  371 VALANILDMYYEDLYTLPDKQLLCGAGFANFTNLSKN-KRISSLLKILTISIASGIFLVA
Fve XP_004295562.1  361 VGLADIIDMYYEDLYTLPDKELSCGD-IASFTNISNK-KTGSFLLKLLSVSLASGILLVA
Ppe EMJ12593.1       335 VGLADIIDMYYEDLYTMPDKQLSCGV-VANSTNLSNK-KMGSFLLRMLSVTLASGIFLIT
Consensus           421 ..*....*....*..... ... ...  . . ..  . ..  ..................

Ppa XP_001764411.1  430 IG--GALLAGFLSSRRARLGQMDAPIESIFDMAFSQKLREIGRSD----IVDEQMDSVNQ
Sbi XP_002451363.1  417 VTGFARLCWPRSFRDKMLFNEISNVSSSQNYCSDIKSLDNSEIQAYCTSLVKKMKDSYGC
Osa BAD07831.1       414 VGAIAQLCWPQSLKDKRLATVSSCVSSSQSYCSDIHSLDSSELQAYCVSVVEKIKDSFGC
Ata EMT06249.1       298 VGVVAQLCWPQSLKGKRLFMGSSPTSSSQSYCSDVRSLDNSEVRGYCISVVKKIKDSCGC
Tur EMS59110.1       345 VGVVAQLCWPQSLKGKRLFMGSSPTSSSQSYCSDVHSLDNSEVRGYCISVVKKIKDSCGC
Sly XP_004234099.1  434 IRILGQRYLPYLPIRKHYHPGVNPVNSSDMISIQPQSMESCKLEDYCVSVVRRIKEFYCW
Cru EOA37571.1       413 VSAAAQFCFPQ-KSERKYPGRSQDILWSENELLSHQSSDSSKLDSFCGLLVNKLKDAYCW
Ath NP_174175.2      418 VSAAAQFCLPQ-KSERKYPGKRQEILWSESELLSHQSSDSSELDSFCGLLVNKLKDAYSW
Aly XP_002893534.1  420 VSAAAQFCLPH-KSERKYPGKRQDILWLESELLSHQSSDFSELDSFCGLLVNKLKDAYSW
Csa XP_004134942.1  433 IGAVGQRFMSRVRLPGRYSAEQPITSLDGLSSVKDQAIEAAKLEEYCISVVKIIKDAFGW
Gma XP_003535421.1  421 ISALGQFCLPWLCKERKHSVEHRSLPSSEVDVAINDFFDTQKLEEYCVLAIAKAKGTFGW
Gma XP_003541453.1  417 ISALGQFCLPWLCKERKHSVEHRSLPSSEVNVAMNDFFDTKKLEECCVLAIAKVKDTFGW
Mtr XP_003593674.1  428 ISALGQFCLPRLSKERKHTVEHRSLLTSEVNV-MHDFLDTTKVEEFCVSAVAKLKNAYGW
Car XP_004485849.1  452 ISALGQFWLPRLSKERKHPVEHRSLPTSEVNI-MHDILDATKVEEFCVPAVAKVKDAFGW
Rco XP_002522507.1  418 ISAFSRFYFPHLHKQQTYNQEHRSPPSSEIEHSVNESLHAEKLQEFCILIVKKIKDAFGW
Tca EOY28124.1       429 ISTLSQFSFPHLHKGGKYPGGSRSPPSSIIESTTIQSVHAEKLEAFCILVIQKIKDAFGW
Vvi CBI30631.3       431 INVLGRLYFPRVNEVGKGPGNQPSLLPSEINCVQHNSLETTKMEAFCISIVKKIKDAFGW
Vvi XP_002273236.2  430 INVLGRLYFPRVNEVGKGPGNQPSLLPSEINCVQHNSLETTKMEAFCISIVKKIKDAFGW
Fve XP_004295562.1  419 ISALGQFGLPYLHKGGKYLGERRSIPSSEVDSALNQCSDTVKLEAFCESVVKKVKDSLGW
Ppe EMJ12593.1       393 ISALGQLGFPYPHKGAKYLREDRSFPSSEVDGALYQSVDAVKLEAFCVSLVKKIKDALGW
Consensus           481 .......... . . ..       .. ....     ......... ..............
```

```
Ppa XP_001764411.1   484 QSEVKADH-----------------------TEAIVKETTEGAG----------------
Sbi XP_002451363.1   477 PGEVMVDAHIGAWVGELPNCFKEINSEDTAASGYFQHPDTLSQENQSQSVPINIKM-SNL
Osa BAD07831.1       474 TGDLMVDANIGAWVGELPEFFKGINCDSHDDYVDIQSTGAISQGEQPSLVSSPIKMSSHL
Ata EMT06249.1       358 PGDIVVDENIGAWIGELPDCFKAINFGDNAASDDAQYSRTVIKENKNPLVSTPTGMTSHL
Tur EMS59110.1       405 PGDIVVDENIGAWIGELPDCFKAINLGDNAASDDAQYSCTVIKENKNPLVSTPTEMTSHL
Sly XP_004234099.1   494 PGDVVMGSGSSAWIGELPIYLKN---EMDSEVLDINSSSTPSEG----------------
Cru EOA37571.1       472 VGEITLESSIGAWIGEVPDYLKETS-RAKSVDDPIVSSSSLLEK----------------
Ath NP_174175.2      477 VGEITLESSIGAWIGEVPDYLKETS-RAKSVEDHIVTSSSLLEI----------------
Aly XP_002893534.1   479 VGEITLESSIGAWIGEVPDYLKETS-RAKSVEDHIVTSSSLLEK----------------
Csa XP_004134942.1   493 HGDVRTGKRVGAWIGDAPDYLTVV--ESDIRSEDAPSGTIGEEN----------------
Gma XP_003535421.1   481 SGEIKVEDGIGVWIGELPAYLR----------GEGVDTLST-SEN---------------
Gma XP_003541453.1   477 LGEIKVEDGIGVWIGELPAYLR----------GEGVDTLST-SEN---------------
Mtr XP_003593674.1   487 SDEIKVEDGIGAWIGELPVYLR----------GEGVDTLSTSSED---------------
Car XP_004485849.1   511 SDEIKVD-GIGAWIGELPAYLR----------GDGVDTLSTSSED---------------
Rco XP_002522507.1   478 PGDIITETSNGAWIGEIPNYLKAM--GEYDSSKEDSSTSDPIEK----------------
Tca EOY28124.1       489 PGDIATETDLSAWIGEMPNYLKVPH-QANSSNEDDSASSADVGK----------------
Vvi CBI30631.3       491 SGEIMAESSVGAWTGDLPIYLRVD--KANHSGEDISNGTALLQR----------------
Vvi XP_002273236.2   490 SGEIMAESSVGAWTGDLPIYLRVD--KANHSGEDISNGTALLQR----------------
Fve XP_004295562.1   479 PGEITLERNVGAWTGKVPDYLRMAV-EDHLNNEDLSTISVPLDK----------------
Ppe EMJ12593.1       453 PGEIKMETDVGAWTGKVPNYLRMVV-EDSSNREDMSTFSSSSEK----------------
Consensus            541  ... ..  ......... ...          ... . .. ..

Ppa XP_001764411.1   505 -LDEQSEPVIEQPAAEQVYVSLQ-----FPSQNTLISTSGPALTVGGFLY--NQLSLSMS
Sbi XP_002451363.1   536 DQNDRTQETLQNIASFHVVISVEGKVVGFQPTNRPAVNHWATNPLATLLYQGRSLSPGVK
Osa BAD07831.1       534 EQNDDTQETLQNIASFQVVMSERGKVVGFQPTNRLAVNHWATNPLTKLLYEGRKISPAFL
Ata EMT06249.1       418 EQNDNTQESLQNIASFQVVMSEEGKLVGFQPTSRLAVNHWAKNPLAALLYEGRKLSPAFL
Tur EMS59110.1       465 EQNDNTQESLQNIASFQVVMSEEGKLLGFQPTSCLAVNHWAENPLAALLYEGRKLSPAFL
Sly XP_004234099.1   535 -IEDEMKAAGEDIASYQIVLSGDGKIAGFQPTSRVAESQWEANPLVKELYGTEKLSPDLI
Cru EOA37571.1       515 -LNEDAKASAQDIATYQVVLSPEGKIIGFQPTSRVAVNHWAANPLARELYEGKKLSPGLI
Ath NP_174175.2      520 -LNEDAKASAQDIATYQVVLSSEGKIIGFQPTSRVAVNHWAANPLARELYGGKKLKPGLI
Aly XP_002893534.1   522 -LNEDAKAAAQDIATYQVVLSAEGKIIGFQPTSRVAVNHWAANPLAKELYGGKKLKPGLI
Csa XP_004134942.1   535 -IDE-VKASAQDIASYQVVLTTEGKIVGFQPTSRVAVNYWAANPLAKQLYGGKNLSPGFI
Gma XP_003535421.1   515 -IDADAKVSIQDIASYQVVFSSEGKIVGFQPLSRVAVNHWSVNPLARELYGGKKLSPSII
Gma XP_003541453.1   511 -IDADAKVPIQDIASYQVVFSSERKIVGFQPLSRVAVNHWAANPLARELYRGKKLSPSLI
Mtr XP_003593674.1   522 -IDADAKVSMQDIASYQVVFSSEGKIVGFQPLSRVAVNQWADNPLAKELYGGKKLSPGIV
Car XP_004485849.1   545 -IDADVKVSMQDIASYQVVFSSEGKIVGFQPLSRVAVNQWADNPLARELYGGKKLSPGII
Rco XP_002522507.1   520 -LDEDMKSSAQDIASYQVVLSTDGKIVGFQPTSRIGVNHWAANPLARELYAGRKLSPGLV
Tca EOY28124.1       532 -IDDDIKSSVQDVASYQVILSTEGKIVGFQPTSRVGVNHWAVNPLAKELYGGRKLSPGLI
Vvi CBI30631.3       533 -SDEEKKTSTQDIASYQVVLSVDGDIVGFQPTSRVAVNNWAVNPLAKELYKGKKLSPGLF
Vvi XP_002273236.2   532 -SDEEKKTSTQDIASYQVVLSVDGDIVGFQPTSRVAVNNWAVNPLAKELYKGKKLSPGLF
Fve XP_004295562.1   522 -IDEDLKASAQDIASYQVVLSADGKIVGFQPLSRAAVNHWAANPLSKELYGGKKLSPGLM
Ppe EMJ12593.1       496 -IDEDLKASAQDIASYQVVLSTDGKIVGFQPLSSVAVNHWAANPLAKELYRGRKLSPGLT
Consensus            601  .... . .....*....... ......*..................**..........

Ppa XP_001764411.1   557 RWRVCRCD---------PYSRDDCEV-------SGF-----VSG--------------
Sbi XP_002451363.1   596 DPSLMESLLVIQEWGSALTAIWKCIWVHGINGFCAY-----GSLSSIQYSAP-------
Osa BAD07831.1       594 EPRLRISR---------PAKVVPVELLMSVNPESFF-----ALVRPVQDPC--------
Ata EMT06249.1       478 EPRLKISR---------PAKVVPIELLMSVNSESFF-----ALARPVQDPC--------
Tur EMS59110.1       525 EPRLKISR---------PAKVVPIELLMSVNSESFF-----ALARPVQDPC--------
Sly XP_004234099.1   594 EQGLKRSR---------GSDAVVLELLMSTNPQSSF-----ALVRPVTENSR-------
Cru EOA37571.1       574 EPGLKSRP---------PTKVVVLELLMSVNSDRPF-----ALVRPILPQ---------
Ath NP_174175.2      579 EPGLKSHP---------PKKVVVLELLMSVNSDRPF-----ALVRPILPQ---------
Aly XP_002893534.1   581 EPGLKSHP---------PKKVVVLELLMSVNSDRPF-----ALVRPILPQ---------
Csa XP_004134942.1   593 ETGLRIER---------PNEVVVIELLMSVKTDAFF-----ALARPVYY----------
Gma XP_003535421.1   574 EPGLKVSL---------PEKVIVVELLMSINPDAYF-----ALARPFR-----------
Gma XP_003541453.1   570 EPGLKVPL---------PEKVIVVELLMSINPDAYF-----ALARRFR-----------
Mtr XP_003593674.1   581 EPGLRIHL---------PKEVTVVELLVSVKPDAYF-----ALARPYQ-----------
Car XP_004485849.1   604 EPGLRVLL---------PEKVIVVELLMSVKPDVYF-----AMARPYQ-----------
Rco XP_002522507.1   579 EPGLKIRP---------PSEIVVIELLMSLWADWHYRNLTASLGGSIHECAPGSKFIHL
Tca EOY28124.1       591 ERGLKIRL---------PNEIVVIELLMSVNPDSCF-----ALARPIQ-----------
Vvi CBI30631.3       592 ETGLKIPR---------PNEVVIIELLMSVNSDACF-----ALARPVQ-----------
Vvi XP_002273236.2   591 ETGLKIPR---------PNEVVIIELLMSVNSDACF-----ALARPVQ-----------
Fve XP_004295562.1   581 EPGLKVQC---------PDEIAVIELLMSVKPDASF-----AFARPVR-----------
Ppe EMJ12593.1       555 EPGLKVQR---------PNEVAVIELLMSVKPDACF-----ALARPVR-----------
Consensus            661 ......            .  ........... .  .     ......
```

B

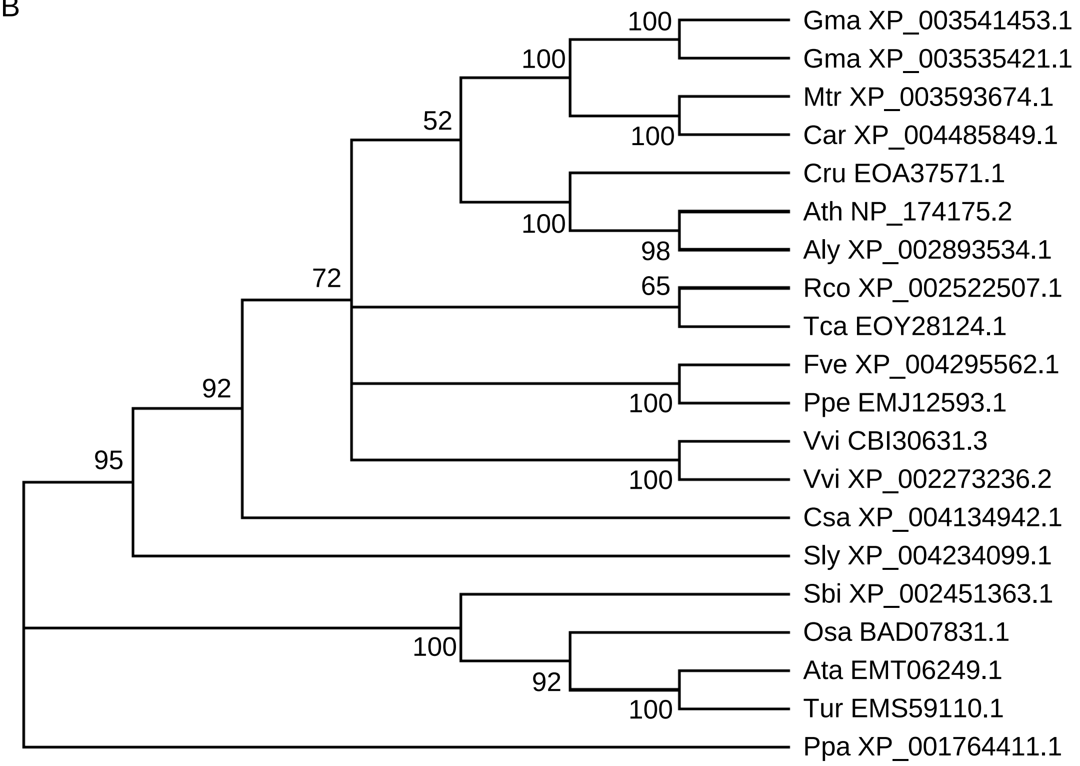

Supplementary Figure S4. Phylogenetic analysis of ANU10 and its putative orthologs. (A) Alignment of the amino acid sequences of the Arabidopsis ANU10 protein and its orthologs in other plant species. Identical and closely related residues that are conserved in >50% of the sequences are shaded black and grey, respectively. Asterisks and dots indicate positions with totally identical or similar residues, respectively, among the orthologs. Numbers indicate residue positions. Amino acids drawn in red and green correspond to the chloroplast transit peptides as predicted by ChloroP1.1 and the transmembrane domains as predicted by SOSUI, respectively. Protein sequences were retrieved from NCBI. The multiple sequence alignment was first made using T-Coffee, subsequently refined using MUSCLE and finally shaded with BOXSHADE 3.21. (B) Unrooted tree showing the evolutionary history of ANU10 and homologous proteins. Analysis of 20 homologs was carried out with MEGA5 following the Neighbor-Joining method. The evolutionary distances were calculated using the Poisson correction method. A bootstrap consensus tree was inferred from 1000 replicates. Numbers correspond to bootstrap percentages. Branches corresponding to partitions reproduced in less than 50% bootstrap replicates were condensed. Plant species and identities (%) between ANU10 and each of its orthologs are: Ppa, *Physcomitrella patens* (18.06%); Sbi, *Sorghum bicolor* (29.46); Osa, *Oryza sativa* (32.00); Ata, *Aegilops tauschii* (28.17); Tur, *Triticum urartu* (28.95); Sly, *Solanum lycopersicum* (45.51); Cru, *Capsella rubella* (85.76); Ath, *Arabidopsis thaliana*; Aly, *Arabidopsis lyrata* (89.98); Csa, *Cucumis sativus* (48.18); Gma, *Glycine max* (52.83 for XP_003541453.1 and 52.97 for XP_003535421.1); Mtr, *Medicago truncatula* (52.15); Car, *Cicer arietinum* (49.85); Rco, *Ricinus communis* (50.94); Tca, *Theobroma cacao* (52.47); Vvi, *Vitis vinífera* (52.55 for CBI30631.3 and 52.39 for XP_002273236.2); Fve, *Fragaria vesca* (54.84) and Ppe, *Prunus persica* (52.58).

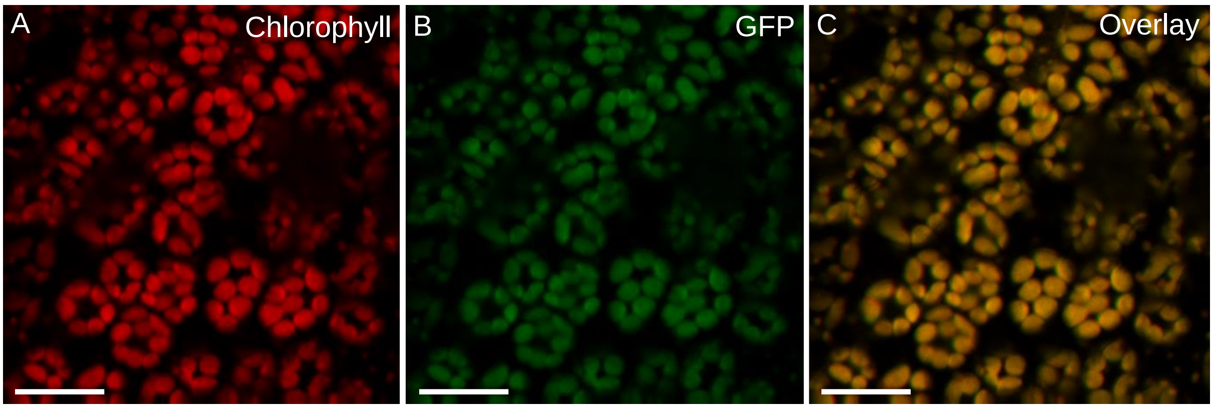

Supplementary Figure S5. Subcellular localization of the ANU10:GFP fusion protein in the *anu10-1* *ANU10*$_{pro}$*:ANU10:GFP* transgenic line. Confocal micrographs of the subepidermal layer of mesophyll cells showing (A) chlorophyll autofluorescence of the chloroplasts, (B) GFP fluorescence and (C) an overlay of the chlorophyll and GFP signals. Pictures were taken from first-node leaves collected 16 das. Scale bars indicate 20 µm.

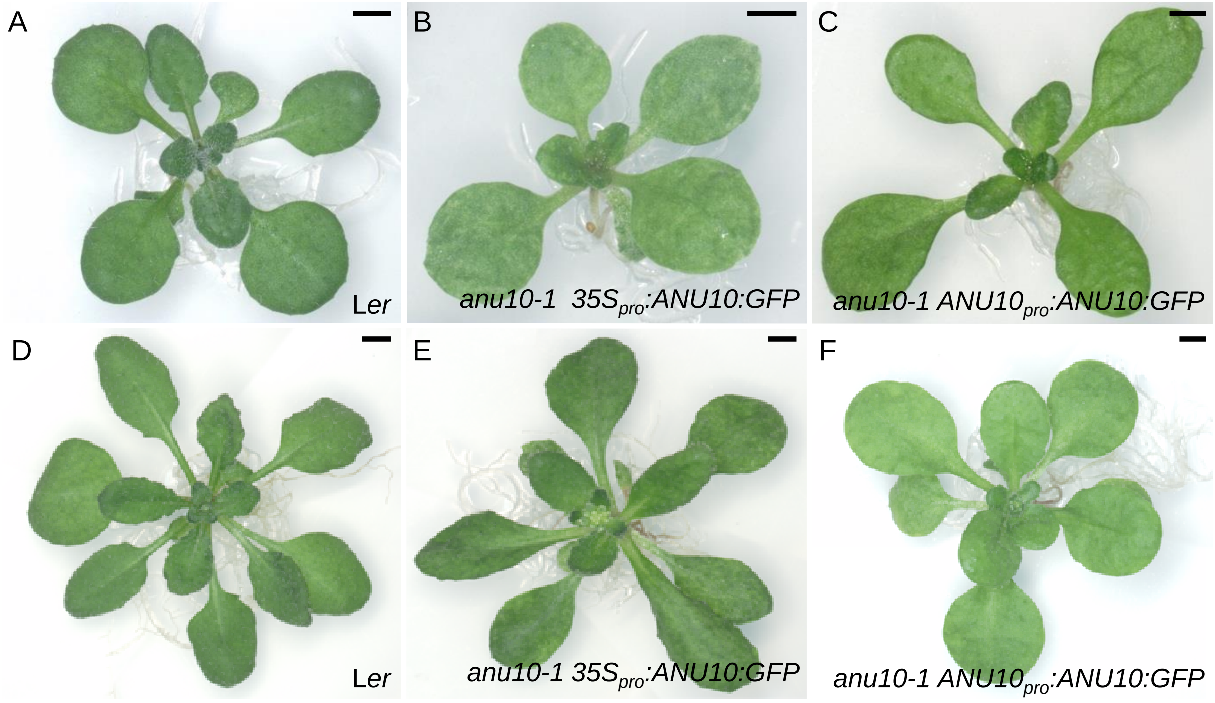

Supplementary Figure S6. Phenotypic rescue of *anu10-1* by the ANU10:GFP fusion protein. Pictures were taken (A-C) 16 and (D-F) 21 das. Scale bars indicate 2 mm.

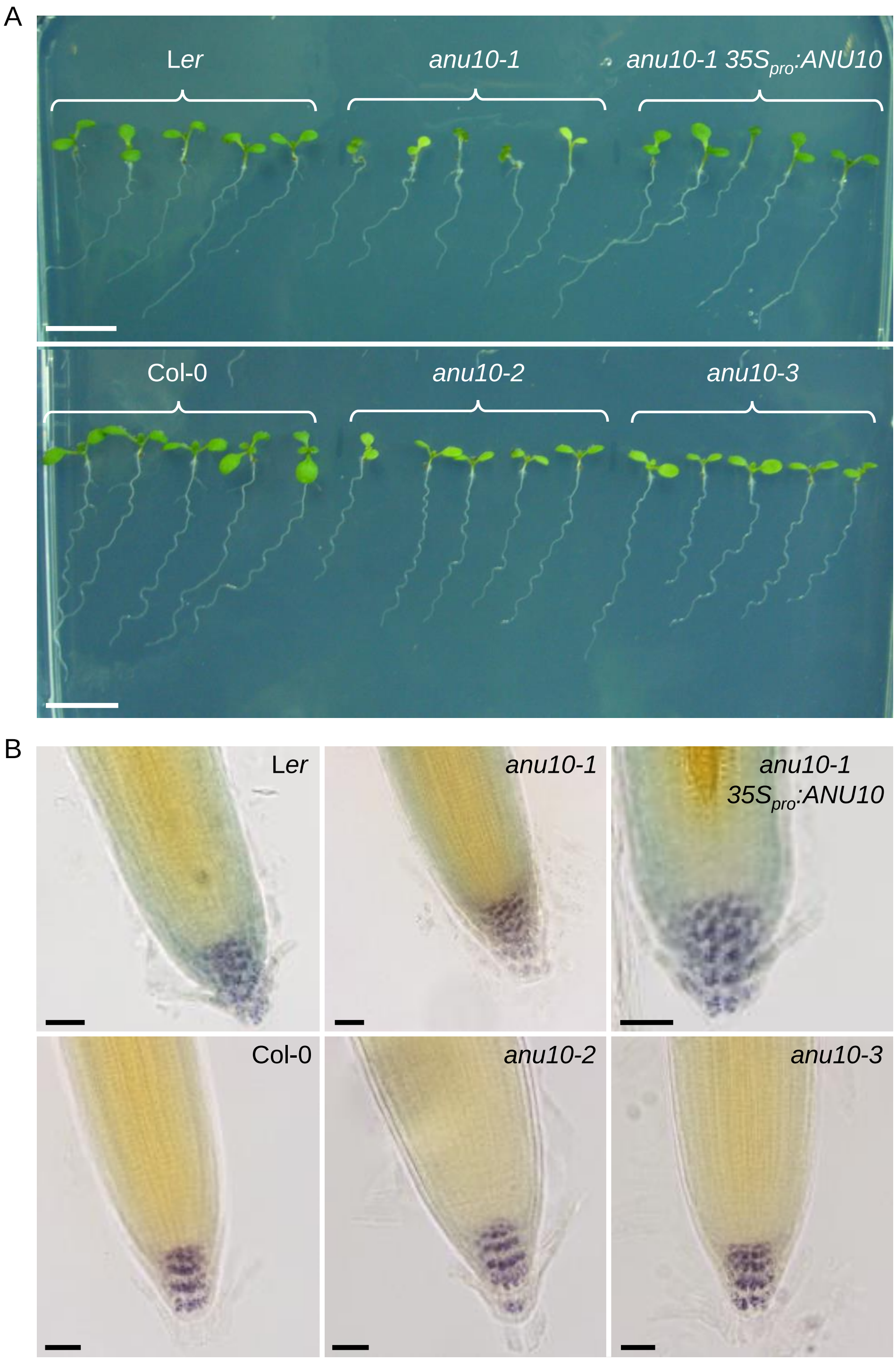

Supplementary Figure S7. Growth and starch content of *anu10* roots. (A) Plants grown on vertically oriented agar plates. (B) Lugol staining of root apices. Pictures were taken (A) 7 and (B) 9 das. Scale bars indicate (A) 1 cm and (B) 100 µm.

Supplementary Table S1. Primer sets used in this work

Purpose	Oligonucleotide name(s)	Oligonucleotide sequences (5'→3')	
		Forward primer (F)	Reverse primer (R)
Linkage analysis	AthZFPG_F/R	TTGCGTTTCCACATTTGTTTAAAC	AGAAATGGGTCAATTCACATGTA
	SNP10026_F/R	CAAGCCCTCTTATGCTTTAGAAA	ACTATTGACTTCGTGTTAACAAAG
	nga392_F/R	TTGAATAATTTGTAGCCATGTTAAT	GGGGTGTTAAATGCGGTGTTC
	F3M18_F/R	GCTGTCAATTTAGGGCATTGA	AAAAGTTGGTTTGAAAAAGTTGAGA
	F1K23_F/R	CCTGTCAAGGGGTCGTAGTC	TTGTGCCTCATCTTCTCGTG
	cer479911_F/R	CAAAATAGAACCAACACCGTAAA	ATTCACTTTCCAATAGATCACTC
	SO392_F/R	GACGATATCTTCCTGCGAAAGA	TGTAAAGAGAAGCAGAGTGTCG
ANU10 sequencing	ANU10_F1/R1	TGGGGAAATACGGTTTTGAG	CGAGATGCTGATATGTTGTGTG
	ANU10_F2/R2	AGGACTGGCAATTGGTTCTG	TTTCGCCGACGAAGAAGTAT
	ANU10_F3/R3	AGAGCGGAGATGCATATAACT	TTATTTTGGAGACATGTTATGAG
	ANU10_R4		GGGAATTGATACTGATAGGTTC
Topo and Gateway cloning	ANU10cds_F/R	CACCATGGCTTCCATTTCTACTGCT	TTGTGGCAATAATGGTCTGAC
	ANU10cds_F2/R2	GCAGCTGTGGAACTTCCAGC	ACAGTACCAACGAGACTGAGC
	ANU10cds_F3	ATCCTTTAGCTAGAGAACTTTATG	
	ANU10pro_F/R	GGGGACAAGTTTGTACAAAAAAGCAGGCT<br>TTGCTGCTCTGCATAGACACA	GGGGACCACTTTGTACAAGAAAGCTGGGT<br>GAATGGAAGCCATTGACGAAC
	ANU10pro_F2	CGCAGATACGGAATCATTTTTCT	
	ANU10cds-pro	TCTGTTCGTCAATGGCTTCCATTTCTACTGCTT	
	ANU10pro-cds	ATGGAAGCCATTGACGAACAGATAAAAATCACAGA	
	M13_F/R	GTAAAACGACGGCCAG	CAGGAAACAGCTATGACCATGATT
qRT-PCR	18S RRNA_F/R	GGTGGTGGTGCATGGCCGTTCTT	GACGGGCGGTGTGTACAAAGGGC
	LHCB1_F/R[1]	GCCTTCGCTACCAACTTCGTC	AACCGGATACACACAACTCGATC
	LHCB2_F/R	CTCAAAGCATCTGGTACGGA	CGGTTAGGTAGGACGGTGT
	LHCB3_F/R	AGTACACTATGGGAAATGATCT	GTGAGGTAAGACGGAGTTTG
	LHCB5_F/R	GGTATGGTCCTGACAGGAG	CTTCTTTCCAAGACCAAATGG

qRT-PCR	HEMA1_F/R[2]	GTGAGCTCTCTGCTTCTTCTGATTCTG	CTGCTTCTTTCCTTTGTATATCGATCAGCT
	ORE1_F/R[3]	CTTACCATGGAAGGCTAAGATGGG	TCGGGTATTTCCGGTCTCTCAC
	accD_F/R[4]	GCTAAGTAAAGCAATGGATAGTTT	CGAATGTCCTTGGAGCTAACTAA
	rbcL_F/R[5]	GTGTTGGGTTCAAAGCTGGT	CATCGGTCCACACAGTTGTC
	psbA_F/R[5]	GAGCAGCAATGAATGCGATA	CCTATGGGGTCGCTTCTGTA
	rrn16_F/R[5]	CGGTATCTGGGGAATAAGCA	GATTTGACGGCGGACTTAAA
	rrn23_F/R[5]	GGGCGACTGTTTACCAAAAA	TTACCCGACAAGGAATTTCG
	atpB_F/R[5]	GAGCTCGTATGAGAGTTGGT	ACCCAATAAGGCGGATACCT
Genotyping of mutant and transgenic plants	LB1_F[a]	GCCTTTTCAGAAATGGATAAATAGCCTTGCTTCC	
	N831342_F/R	TGTGTCAACCAGATTGGTCAG	TTGCTAGGTTCATTCCCACAC
	N828696_F/R	TTTACTGTATTGCCGAATCGC	AGCGAAGTTAACCTCTCGAGG
	35S_F[b]	GACAGTGGTCCCAAAGATG	
	GFP_R[c]		TATGTTGCATCACCTTCACCCT
	GUS_R[d]		CACAAACGGTGATACGTACACT

[a-d]Each of these oligonucleotides was used together with: [a]N831342_R and N828696_R to genotype the *anu10-2* and *anu10-3* mutants; [b]ANU10cds_R2, [c]ANU10cds_F2 or ANU10cds-pro, and [d]ANU10pro_F2 to genotype transgenic plants putatively carrying the [b]*35S$_{pro}$:ANU10*, [c]*35S$_{pro}$:ANU10:GFP* or *ANU10$_{pro}$:ANU10:GFP*, and [d]*ANU10$_{pro}$:GUS* transgenes.[1]Adhikari *et al.* (2011), [2]Mochizuki *et al*. (2008), [3]Rauf *et al*. (2013), [4]Hricová *et al*. (2006), [5]Gao *et al*. (2011).

Supplementary Table S2. Transmembrane domains and chloroplast transit peptides in the ANU10 protein and its putative orthologs

Organism	Protein[a]	Protein length (aa)	Transmembrane domains[b]		Chloroplast transit peptides[c]	
			Number	Coordinates	Presence	Length (aa)[c]
Physcomitrella patens	XP_001764411.1	579	1	417-439	No	-
Sorghum bicolor	XP_002451363.1	642	0	-	Yes	59
Oryza sativa	BAD07831.1	630	1	400-422	Yes	45
Aegilops tauschii	EMT06249.1	514	2	2-24, 284-306	No	-
Triticum urartu	EMS59110.1	561	1	331-353	No	-
Solanum lycopersicum	XP_004234099.1	631	1	416-438	Yes	46
Capsella rubella	EOA37571.1	609	1	396-418	Yes	34
Arabidopsis thaliana	NP_174172.2	614	1	421-443	Yes	37
Arabidopsis lyrata	XP_002893534	616	1	404-426	Yes	55
Cucumis sativus	XP_004134942.1	627	0	-	Yes	50
Glycine max	XP_003535421.1	607	1	408-430	Yes	38
Glycine max	XP_003541453.1	603	1	404-426	Yes	34
Medicago truncatula	XP_003593674.1	614	0	-	Yes	63
Cicer arietinum	XP_004485849.1	637	1	436-458	Yes	83
Ricinus communis	XP_002522507.1	628	1	403-425	Yes	54
Theobroma cacao	EOY28124.1	624	0	-	Yes	46
Vitis vinifera	CBI30631.3	666	1	415-437	Yes	45
Vitis vinifera	XP_002273236.2	624	1	414-436	Yes	45
Fragaria vesca	XP_004295562.1	614	1	403-425	Yes	56
Prunus persica	EMJ12593.1	588	0	-	No	-

[a]GeneBank accession number. [b,c]Predictions shown in this table were obtained from [b]SOSUI and [c]ChloroP 1.1.

Supplementary Data References

Adhikari ND, Froehlich JE, Strand DD, Buck SM, Kramer DM, Larkin RM. 2011. GUN4-porphyrin complexes bind the ChlH/GUN5 subunit of Mg-Chelatase and promote chlorophyll biosynthesis in Arabidopsis. *Plant Cell* **23**, 1449-1467.

Gao ZP, Yu QB, Zhao TT, Ma Q, Chen GX, Yang ZN. 2011. A functional component of the transcriptionally active chromosome complex, Arabidopsis pTAC14, interacts with pTAC12/HEMERA and regulates plastid gene expression. *Plant Physiology* **157**, 1733-1745.

Hricová A, Quesada V, Micol JL. 2006. The *SCABRA3* nuclear gene encodes the plastid RpoTp RNA polymerase, which is required for chloroplast biogenesis and mesophyll cell proliferation in Arabidopsis. *Plant Physiology* **141**, 942-956.

Mochizuki N, Tanaka R, Tanaka A, Masuda T, Nagatani A. 2008. The steady-state level of Mg-protoporphyrin IX is not a determinant of plastid-to-nucleus signaling in *Arabidopsis*. *Proceedings of the National Academy of Sciences of the United States of America* **105**, 15184-15189.

Rauf M, Arif M, Dortay H, Matallana-Ramirez LP, Waters MT, Gil Nam H, Lim PO, Mueller-Roeber B, Balazadeh S. 2013. ORE1 balances leaf senescence against maintenance by antagonizing G2-like-mediated transcription. *EMBO Reports* **14**, 382-388.
